# Japanese Society of Anxiety and Related Disorders/Japanese Society of Neuropsychopharmacology: Clinical practice guideline for social anxiety disorder (2021)

**DOI:** 10.1002/npr2.12365

**Published:** 2023-08-25

**Authors:** Satoshi Asakura, Naoki Yoshinaga, Hisashi Yamada, Yutaka Fujii, Nobuyuki Mitsui, Yoshihiro Kanai, Takeshi Inoue, Eiji Shimizu

**Affiliations:** ^1^ Japanese Society of Anxiety and Related Disorders Tokyo Japan; ^2^ Japanese Society of Neuropsychopharmacology Tokyo Japan; ^3^ Department of Psychiatry, Hokkaido University Graduate School of Medicine/Health Care Center Hokkaido University Sapporo Japan; ^4^ School of Nursing, Faculty of Medicine University of Miyazaki Miyazaki Japan; ^5^ Department of Neuropsychiatry Hyogo College of Medicine Nishinomiya Japan; ^6^ Department of Psychology and Behavioral Sciences, Faculty of Human Sciences Tohoku Gakuin University Sendai Japan; ^7^ Department of Psychiatry Tokyo Medical University Tokyo Japan; ^8^ Department of Cognitive Behavioral Physiology, Graduate School of Medicine Chiba University Chiba Japan

**Keywords:** anxiety disorders, Japan, practice guidelines, social anxiety disorders, treatment

## Abstract

The development of clinical practice guidelines for social anxiety disorder began in March 2018 when the Joint Clinical Practice Guideline Development Committee for Anxiety and Obsessive–Compulsive Disorders was formed by the Japanese Society of Anxiety and Related Disorders and Japanese Society of Neuropsychopharmacology to jointly develop guidelines for anxiety and obsessive–compulsive disorders. Based on the universal concept of evidence‐based medicine, three clinical questions (CQs) about pharmacotherapy and psychotherapy were developed for clinical guidelines for social anxiety disorder, panic disorders, and obsessive–compulsive disorder in accordance with the Minds “Manual for Guidelines Development 2017 by the Japan Council for Quality Health Care: CQ1—“What is the recommended pharmacotherapy for social anxiety disorder in adults?”; CQ2—“What is the recommended psychotherapy (psychological intervention) for social anxiety disorder in adults?”; and CQ3—“What are the recommendations regarding monotherapy and combination therapy for social anxiety disorder in adults in terms of pharmacotherapy and psychotherapy (psychological interventions)?” Summarized recommendations for social anxiety disorder in adults are selective serotonin reuptake inhibitors and serotonin‐norepinephrine reuptake inhibitor for CQ1, cognitive behavioral therapy for CQ2, and there are no recommendations regarding monotherapy and combination therapy for CQ3. These were answered by considering the balance between benefits and harms based on systematic reviews of each. The aim of this brief guideline for the standard‐of‐care (i.e., medical treatment) for social anxiety disorder in adults (18 years and older) was to help “shared decision making,” in which medical professionals, including physicians, and patients share scientific evidence to decide on a course of treatment.

## Abstract 2

The aim of this brief guideline for the standard‐of‐care for social anxiety disorder in adults was to help “shared decision making,” in which medical professionals, including physicians, and patients share scientific evidence to decide on a course of treatment.

## Graphical abstract

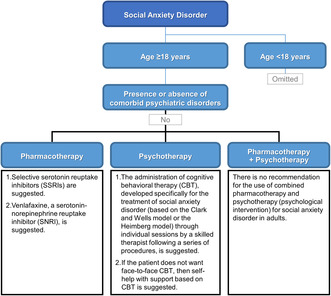

## PREFACE

The development of clinical practice guidelines for social anxiety disorder began in March 2018 when the Joint Clinical Practice Guideline Development Committee for Anxiety and Obsessive–Compulsive Disorders was formed by the Japanese Society of Anxiety and Related Disorders and Japanese Society of Neuropsychopharmacology to jointly develop guidelines for anxiety and obsessive–compulsive disorders. Based on the universal concept of evidence‐based medicine, three clinical questions (CQs) about pharmacotherapy and psychotherapy were developed for clinical guidelines for social anxiety disorder, panic disorders, and obsessive–compulsive disorder in accordance with the Minds Manual for Guidelines Development 2017 by the Japan Council for Quality Health Care. These were answered by considering the balance between benefits (i.e., treatment response and symptom improvement) and harms (i.e., dropout from treatment) based on systematic reviews of each. The aim of this brief guideline for the standard‐of‐care (i.e., medical treatment) for social anxiety disorder in adults (18 years and older) was to help “shared decision making,” in which medical professionals, including physicians, and patients share scientific evidence to decide on a course of treatment.

## BASIC POLICY OF GUIDELINE DEVELOPMENT

This guideline was developed to provide current evidence‐based knowledge and support for the treatment of anxiety in clinical situations. This guideline is not intended to dictate treatment. It is necessary for therapists to devise treatment at their own discretion on a case‐by‐case basis, without being bound by the guideline.

## Disclaimer

As stated in the basic policy, this guideline is not intended to dictate treatment. Therefore, mere compliance with the guidelines does not exempt a person from liability for negligence. Deviation from the guideline cannot be regarded as negligence. The content of the guideline is not a basis for medical litigation.

## GUIDELINE SUMMARY (SEE FIGURE 1 FOR A PRACTICAL ALGORITHM)

CQ1: What is the recommended pharmacotherapy for social anxiety disorder in adults?

Recommendation:

1. Selective serotonin reuptake inhibitors (SSRIs) are suggested.

(GRADE 2C, Strength of recommendation “weak”/Certainty of evidence “low”).

2 Venlafaxine, a serotonin‐norepinephrine reuptake inhibitor (SNRI), is suggested.

(GRADE 2C, Strength of recommendation “weak”/Certainty of evidence “low”).

CQ2: What is the recommended psychotherapy (psychological intervention) for social anxiety disorder in adults?

Recommendation:

1. The administration of cognitive behavioral therapy (CBT), developed specifically for the treatment of social anxiety disorder (based on the Clark and Wells model or the Heimberg model) through individual sessions by a skilled therapist following a series of procedures, is suggested.

(GRADE: None, Strength of recommendation “weak”/Certainty of evidence “low”).

Although this therapy can be administered as group therapy (group session), individual therapy is prioritized because of its superiority in terms of clinical‐ and health‐related economic effectiveness.

2 If the patient does not want face‐to‐face CBT, then self‐help with support based on CBT is suggested.

(GRADE: None, Strength of recommendation “weak”/Certainty of evidence “low”).

CQ3: What are the recommendations regarding monotherapy and combination therapy for social anxiety disorder in adults in terms of pharmacotherapy and psychotherapy (psychological interventions)?

Recommendation:

There is no recommendation for the use of combined pharmacotherapy and psychotherapy (psychological intervention) for social anxiety disorder in adults.

The complete original guidelines written in Japanese (published September 1, 2021), which include additional supporting information, is available at the Minds Guideline Library website (https://minds.jcqhc.or.jp/n/med/4/med0458/G0001312).

**FIGURE 1:**
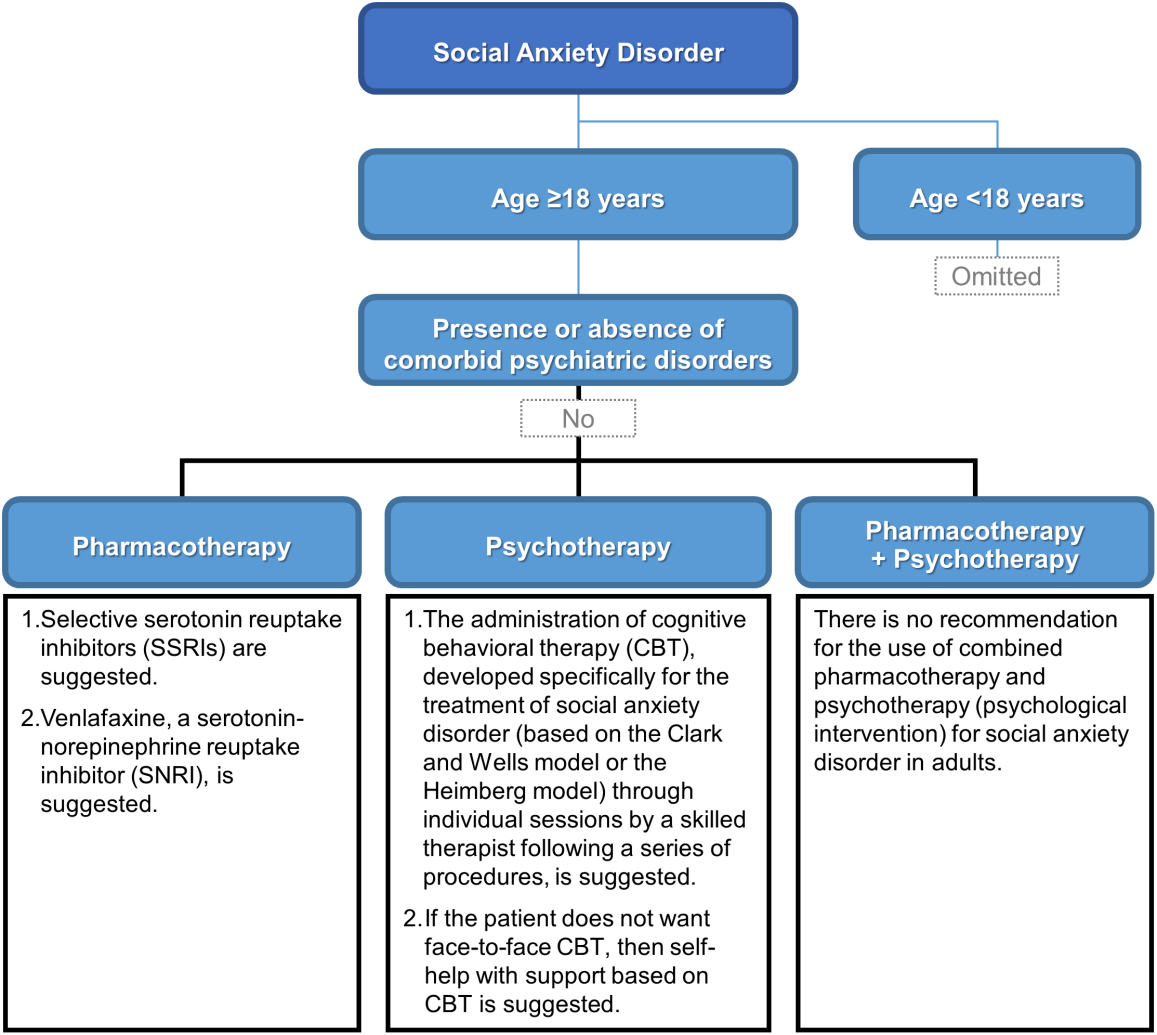
Practice algorithm.

## LIST OF TERMS AND ABBREVIATIONS

### Terminology

Cognitive behavioral therapy (CBT): Psychotherapy that modifies and improves cognition (i.e., the way of thinking about and perceiving events) and behavior to reduce unpleasant feelings and improve social adjustment.

Meta‐analysis: Statistical analysis to integrate and analyze results of multiple studies.

Network meta‐analysis: Statistical analysis for the simultaneous meta‐analysis of three or more intervention groups.

Randomized controlled trial (RCT): A research method that aims to avoid evaluation bias and objectively evaluates treatment effects. Participants are randomly assigned to the intervention group and comparison group for implementation and evaluation.

Selective serotonin reuptake inhibitor (SSRI): Drug that inhibits serotonin reuptake in the brain and enhances serotonin function.

Serotonin‐norepinephrine reuptake inhibitor (SNRI): Drug that inhibits the reuptake of serotonin and norepinephrine in the brain and enhances serotonin and norepinephrine function.

Systematic review: A systematic and comprehensive review of the literature and data analysis of high‐quality studies, such as RCTs, removing as much data bias, such as publication bias, as possible.

### Abbreviations

5‐HT_1A_: 5‐hydroxytryptamine‐1A

ADHD: attention‐deficit/hyperactivity disorder

AGREE: Appraisal of Guidelines for Research and Evaluation

APA: American Psychiatric Association

CANMAT: Canadian Network for Mood Disorders

CBT: cognitive behavioral therapy

CGI: clinical global impression

CI: confidence interval

CPG: clinical practice guideline

CQ: clinical question

DSM: *Diagnostic and Statistical Manual of Mental Disorders*

GABA: γ‐aminobutyric acid

GRADE: Grading of Recommendations Assessment, Development, and Evaluation

ICD: *International Statistical Classification of Diseases and Related Health Problems*

IGL: International Guideline Library

LSAS: Liebowitz Social Anxiety Scale

MAOI: monoamine oxidase inhibitor

NARI: norepinephrine reuptake inhibitor

NGC: National Guideline Clearinghouse

NICE: National Institute for Health and Care Excellence

NNT: number needed to treat

NaSSA: noradrenergic and specific serotonergic antidepressants

PTSD: post‐traumatic stress disorder

QOL: quality of life

RCT: randomized controlled trial

RR: relative risk

RIMA: reversible inhibitor of monoamine oxidase A

SARI: serotonin 2 antagonist and reuptake inhibitor

SMD: standardized mean difference

SNRI: serotonin‐norepinephrine reuptake inhibitor

SPIN: Social Phobia Inventory

SSRI: selective serotonin reuptake inhibitor

WHO: World Health Organization

## ORGANIZATION AND DEVELOPMENT PROCESS

### Development organization

#### Developed by

Japanese Society of Anxiety and Related Disorders and Japanese Society of Neuropsychopharmacology.

#### Guideline management committee

**Table:** 

Eiji Shimizu	Chairperson, Department of Cognitive Behavioral Physiology, Graduate School of Medicine, Chiba University, Chiba‐shi, Chiba, Japan; Psychiatrist; Certified Public Psychologist; Researcher in CBT and RCTs
Takeshi Inoue	Commissioner, Department of Psychiatry, Tokyo Medical University, Shinjuku‐ku, Tokyo, Japan; Psychiatrist; Researcher in psychopharmacology

#### Guideline development group (committee)

**Table:** 

Eiji Shimizu	Chairperson of the Committee

Committee members are listed below:

**Table:** 

Takeshi Inoue	Department of Psychiatry, Tokyo Medical University, Shinjuku‐ku, Tokyo, Japan
Tsukasa Sasaki	Department of Health Education, Graduate School of Education, The University of Tokyo, Bunkyo‐ku, Tokyo, Japan
Hiroaki Kumano	Institute of Applied Brain Sciences, Faculty of Human Sciences, Waseda University, Tokorozawa, Saitama, Japan
Ken Inada	Department of Psychiatry, Tokyo Women's Medical University School of Medicine, Shinjuku‐ku, Tokyo, Japan
Hisato Matsunaga	Department of Neuropsychiatry, Hyogo Medical University, Nishinomiya, Hyogo, Japan
Toshiki Shioiri	Department of Psychiatry, Gifu University Graduate School of Medicine, Gifu‐shi, Gifu, Japan
Satoshi Asakura	Health Care Center and Department of Psychiatry, Graduate School of Medicine, Hokkaido University, Sapporo, Hokkaido, Japan
Hissei Imai	Ohashi Clinic, Takarazuka, Hyogo, Japan
Nozomi Takeshima	Department of Psychiatry, Kitabayashi Hospital, Nagoya, Aichi, Japan
Yu Hayasaka	Tsukuba Psychosomatic Clinic, Tsukuba, Ibaraki, Japan
Toshiaki Baba	Bureau of International Health Cooperation, National Center for Global Health and Medicine, Shinjuku‐ku, Tokyo, Japan

#### Systematic review team

**Table:** 

Satoshi Asakura	Representative, health care center and Department of Psychiatry, graduate School of Medicine, Hokkaido University, Sapporo, Hokkaido, Japan
Hisashi Yamada	Department of Neuropsychiatry, Hyogo Medical University, Nishinomiya, Hyogo, Japan
Yutaka Fujii	Department of Psychiatry, Graduate School of Medicine, Hokkaido University, Sapporo, Hokkaido, Japan
Nobuyuki Mitsui	Department of Psychiatry, Graduate School of Medicine, Hokkaido University, Sapporo, Hokkaido, Japan
Naoki Yoshinaga	School of Nursing, Faculty of Medicine, University of Miyazaki, Miyazaki‐shi, Miyazaki, Japan
Yoshihiro Kanai	College of Liberal Arts, Tohoku Gakuin University, Sendai, Miyagi, Japan

#### External evaluation committee

**Table:** 

Hisanobu Kaiya	Panic Disorder Research Center, Warakukai Medical Corporation, Minato‐ku, Tokyo, Japan
Shin‐ichi Suzuki	Faculty of Human Sciences, Waseda University, Tokorozawa, Saitama, Japan
Yasuyuki Okumura	Initiative for Clinical Epidemiological Research, Machida, Tokyo, Japan
Masatoshi Arizono	OCD Support, Musashino, Tokyo, Japan
Masatomi Ikusaka	Department of General Medicine, Chiba University Hospital, Chiba‐shi, Chiba, Japan

#### Guideline development office

**Table:** 

Eiji Shimizu	Executive Director, Department of Cognitive Behavioral Physiology, Graduate School of Medicine, Chiba University, Chiba‐shi, Chiba, Japan
Takeshi Inoue	Department of Psychiatry, Tokyo Medical University, Shinjuku‐ku, Tokyo, Japan
Ken Inada	Department of Psychiatry, Tokyo Women's Medical University School of Medicine, Shinjuku‐ku, Tokyo, Japan

### Development process

#### Development policy and objectives

In March 2018, the Boards of Directors of both the Japanese Society of Anxiety and Related Disorders and Japanese Society of Neuropsychopharmacology decided to organize a joint development committee for a clinical practice guideline for the treatment of anxiety and obsessive–compulsive disorders. The first meeting was held on March 15, 2018, and a development policy was decided and later approved by the Boards of Directors of the Japanese Society of Anxiety and Related Disorders and Japanese Society of Neuropsychopharmacology. The purpose of this project is to develop treatment recommendations based on a comprehensive review of clinical trials on psychotherapy and pharmacotherapy that were conducted in Japan and overseas and conduct a systematic review. It was also decided that if a latest national or international systematic review is available, then this systematic review could be used to develop recommendations. Because clinical practice for the treatment of anxiety and obsessive–compulsive disorders involves a combination of medical assessment, diagnosis, psychotherapy, pharmacotherapy, psychoeducation, and other non‐pharmacological treatments, including psychiatric rehabilitation, in an integrated manner, we aimed to develop guidelines for clinical practice rather than just a treatment guideline.

The purpose of the clinical practice guideline for social anxiety disorder was to provide recommendations for the treatment of adults (18 years of age and older) with social anxiety disorder, balancing beneficial outcomes (e.g., responsiveness to treatment and symptom improvement) and harmful outcomes (e.g., dropout from treatment) of pharmacotherapy and psychotherapy.

#### Precautions for use

This guideline was developed with the aim of assisting clinical practice and not dictating treatment. Physicians will need to devise treatment plans at their own discretion without being bound by this guideline. Therefore, mere compliance with this guideline during treatment does not exempt a physician from liability for negligence, and deviations from this guideline cannot be regarded as negligence. In other words, the content of this guideline is not a basis for medical litigation.

#### Conflicts of interest (for the past 3 years)

Both individual and organizational conflicts of interest were reported according to the items reported and standard amounts disclosed (financial categories I, II, and III), based on the Japanese Association of Medical Sciences COI Management Guidance on Eligibility Criteria for Clinical Practice Guideline Formulation (released in March 2009). The committee discussed eligibility conditions for appointment of the Chair and Vice‐Chair of the committee and the appointment of committee members and whether or not the right to vote should be granted. It was decided that committee members who fall into financial category III would not have voting rights in the committee and that committee members who fall into financial category II would not be able to assume the office of Chair or Vice‐Chair. Conflicts of interest include the following over the last 3 years: (1) directorships or advisory positions in companies or for‐profit organizations and amounts of their compensation, (2) shareholdings and profits derived from such shares, (3) compensation paid as royalties for patent rights by companies or for‐profit organizations, (4) compensation, such as per diem and lecture fees, paid by companies and commercial organizations for time and effort spent by the researcher in attending meetings (e.g., presentations, advice, etc.), (5) compensation paid by companies or commercial organizations for writing manuscripts, pamphlets, etc., (6) research funding provided under contract by companies and commercial organizations, (7) scholarship (support) endowments provided by a company or a for‐profit organization, (8) endowment courses provided by a company or for‐profit organization, and (9) other compensation (e.g., travel not directly related to research, gifts, etc.). Declarations were reported in writing for the declarant and his/her spouse, first‐degree relatives, and persons with whom the declarant shared income or property interests.

**Table:** 

Takeshi Inoue	Lecture fees (Kyowa Pharmaceutical, Pfizer, Mitsubishi Tanabe Pharma, Otsuka Pharmaceutical, and MSD), endowments (Eisai, Sumitomo Dainippon Pharma, Mitsubishi Tanabe Pharma, MSD, Pfizer, and Meiji Seika Pharma)
Ken Inada	Lecture fees (MSD)
Yoshihiro Kanai	Lecture fees (Advantage Risk Management Co., Ltd.)
Toshiki Shioiri	Lecture fees (Mochida Pharmaceutical and Pfizer)
Hisato Matsunaga	Lecture fees (Meiji Seika Pharma, AbbVie, and Eli Lilly Japan)
Eiji Shimizu	Research funding (Sumitomo Dainippon Pharma)
Satoshi Asakura	Lecture fees (Mitsubishi Tanabe Pharma Corporation)
Masatoshi Arizono	Manuscript fees (Kanematsu Wellness)
There are no conflicts of interest for any other committee members.	

In consideration of academic conflicts of interest, the committee was composed of members from multiple professions, including physicians, certified public psychologists, and others. Non‐members were also included in the committee to avoid bias toward one academic society and ensure the fairness of opinion. For the further consideration of academic conflicts of interest, we will try to include pharmacists and other committee members in the next revision of the guideline.

#### Funding

This guideline was developed by the Japanese Society of Anxiety and Related Disorders and Japanese Society of Neuropsychopharmacology, and both societies provided funding for development of the guideline. No other funding was provided by pharmaceutical or other companies. The funding providers had no influence on content of the guideline.

#### Organizational structure

**FIGURE 2:**
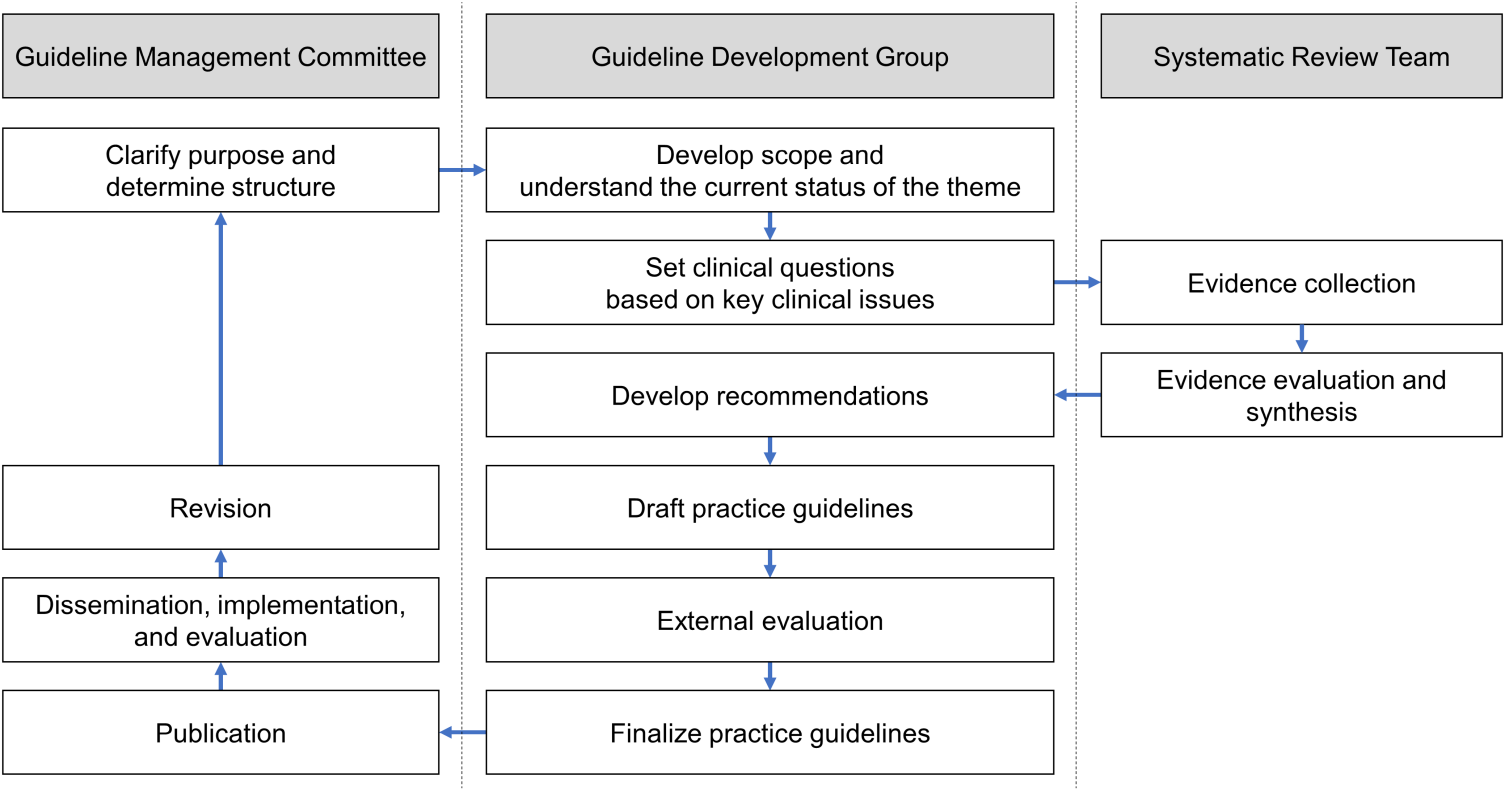
Organizational structure.

The following organizational structure was used to determine recommendations and develop the guideline (Figure 2).

#### Details of the development process

##### Preparation of draft recommendations

In March 2018, a joint committee (Joint Clinical Practice Guideline Development Committee for Anxiety and Obsessive‐Compulsive Disorders) was formed by society members and board members of the Japanese Society of Anxiety and Related Disorders and Japanese Society of Neuropsychopharmacology, and they discussed and decided on the development policy of the clinical practice guideline. After the scope was developed, a systematic review team conducted a comprehensive search of the Japanese and international literature on social anxiety disorder, conducted a systematic review, and developed draft recommendations based on the results.

##### External evaluation

The guideline was evaluated by five external evaluation committee members in October 2019 for applicability and feasibility and for purposes of improving the quality of the guideline and collecting feedback on draft recommendations. An open‐ended question was used, and the evaluation summary included the following: in response to comments on the application of the Grading of Recommendations Assessment, Development, and Evaluation (GRADE) system of network meta‐analysis, the guideline authors considered the evaluation results when making their final recommendation, but they decided not to perform a GRADE evaluation of the network meta‐analysis because of time constraints that prevented them from obtaining the required data for individual studies for direct comparison.

##### Final recommendation decision

At a joint meeting of the Guideline Development Committee on April 19, 2020, 12 of 12 members (including three proxies) voted in favor (approved by more than two‐thirds of the members). The final vote was unanimous, and the recommendations were approved.

Following approval by the Boards of Directors of both societies, the guideline was finalized after collecting and reviewing public comments from patients, families, and other members of the public on the websites of both societies (the call for public comment was announced in May 2021).

## SCOPE

### Intended users and facilities for this guideline

Medical professionals who are involved in the treatment of social anxiety disorder (physicians, certified public psychologists, nurses, mental health social workers, occupational therapists, pharmacists, etc.) and patients with social anxiety disorder and their families and caretakers.

### Potentially indicated clinical settings

- Primary care (including general internal medicine, psychiatry, and psychosomatic medicine).
- Secondary care (specialized psychiatric institutions).

### How to use the guideline

- Standard‐of‐care (treatment) recommendations.

### Basic characteristics of social anxiety disorder

#### Clinical characteristics

According to the American Psychiatric Association (APA) *Diagnostic and Statistical Manual of Mental Disorders* (DSM), the cardinal feature of social anxiety disorder is marked fear or anxiety about one or more social situations in which one may be under the scrutiny of others and a fear that behaving in a certain way or exhibiting anxiety symptoms will be negatively evaluated. Social situations almost always elicit fear or anxiety and are avoided or endured with intense fear or anxiety. The fear or anxiety is disproportionate to the real dangers posed by the social situation and its sociocultural context. The fear, anxiety, or avoidance is persistent, typically lasting 6 months or more. The fear, anxiety, or avoidance causes clinically meaningful distress or impairment in social, occupational, or other important areas of functioning. Furthermore, the fear, anxiety, or avoidance cannot be explained by physiological effects of substances (e.g., drugs of abuse and pharmaceuticals), other medical disorders, or symptoms of other psychiatric disorders, such as panic disorder, dysmorphophobia, or autism spectrum disorder. It is also identified as “performance‐limited” when the fear is limited to speaking or performing an action in public.

Social anxiety disorder is treated with pharmacotherapy and psychotherapy (psychological intervention). The choice of one or both treatments is determined by the patient's availability and preference, taking into account the patient's age, symptoms, and other factors. In Japan, the SSRIs fluvoxamine, paroxetine, and escitalopram are covered by national health insurance. CBT is also available as an insured psychotherapy treatment, which can be provided by a physician or in collaboration with a physician and nurse.

Various pharmacotherapies, including benzodiazepines, are used in clinical practice, and guidelines are needed to determine recommended drugs, including differences in therapeutic efficacy and tolerability, even for SSRIs. In addition to CBT, various other psychotherapies are used, including third‐generation CBT (e.g., mindfulness‐based cognitive therapy, acceptance and commitment therapy, psychodynamic therapy, and Morita therapy). Guidelines are needed to determine the most appropriate psychotherapy. It is also necessary to examine the use of pharmacotherapy or psychotherapy alone and combined.

#### Epidemiological characteristics

According to the DSM, the 12‐month prevalence of social anxiety disorder in adults in the United States is reported to be 6.8%, with a lifetime prevalence of 12.1%. In an epidemiologic study that was conducted by the World Health Organization (WHO) in Japan, the 12‐month prevalence was 0.8%. The median age of onset in the United States is 13 years, with 75% of cases occurring between 8 and 15 years of age. There is a slight gender imbalance, with a slightly higher prevalence among women (odds ratio: 1.5–2.2). Approximately one‐third of patients have other comorbid psychiatric disorders, including other anxiety disorders, depression, mood disorders (e.g., bipolar disorder), and substance use disorders. It has also been noted that social anxiety disorder has a low consultation rate, and approximately 60% of people with social anxiety disorder who do not receive treatment will have symptoms that persist for several years.

#### Overall flow of treatment for social anxiety disorder

Pharmacotherapy and psychotherapy (i.e., psychological interventions) have been shown to be effective treatments for social anxiety disorder in adults. Antidepressants, mainly SSRIs, are effective in the former, and CBT is considered effective in the latter. However, it has not been established which pharmacotherapy or psychotherapy is optimal. The superiority of both treatment modalities or the efficacy of combination therapy has also not been determined.

In this guideline, we examine which pharmacotherapy and psychotherapy methods and combinations thereof are recommended for adult patients with social anxiety disorder.

Therapy changes over time, and it is advisable to always aim for the most updated treatment without waiting for revision of the guideline because situations change with the introduction of new therapeutic drugs and reporting of new evidence. Additionally, changes in terminology and disease classification are expected with the release of the Japanese version of the *International Statistical Classification of Diseases and Related Health Problems*, 11th revision (ICD‐11), by the WHO. Therefore, the information presented herein is not absolute or permanent and should be considered a guide. The guideline is not intended to be a legal standard.

### Matters related to content covered by the clinical practice guideline

Those covered by the guideline (target population, age, and gender):

- Patients with social anxiety disorder (aged over 18 years: adults) of any gender.

Those not covered by the guideline (including excluded populations, clinical conditions, severity of illness, and comorbidities):

- Children and young patients (under 18 years of age) with social anxiety disorder.
- Patients with other comorbid psychiatric disorders (e.g., schizophrenia, bipolar disorder, substance use disorders, developmental disorders, etc.).
- Patients with clinically problematic and unstable concomitant physical illness.
- Women who are pregnant or may become pregnant.
- Patients with organic brain diseases and neurodegenerative diseases.
- Patients at obvious risk of self‐harm/suicide or other harm.
- Patients with obvious intellectual disabilities.

### Matters related to systematic review

#### Searching for evidence

1. Evidence type:
  Existing practice guidelines, systematic review/meta‐analysis articles, and individual study articles were searched in this order of priority. If sufficient evidence was found for the highest‐priority evidence type, then the search ended and proceeded to evidence evaluation and synthesis. All RCTs were included in the search for individual research articles.
2. Database:
  For individual research articles, we used Medline, CINAHL (search for psychotherapy), PsychINFO (search for psychotherapy), PubMed (search for pharmacotherapy), and ICTRP (search for pharmacotherapy). For systematic review/meta‐analysis articles, we used Medline and the Cochrane Library. For existing guidelines, we used the Guideline International Network's International Guideline Library (IGL) and the Agency for Healthcare Research and Quality National Guideline Clearinghouse (NGC). For Japanese‐language literature, we used Ichu‐shi web.
3. Basic search policy:
  The Patient/Population, Intervention, Comparison, and Outcomes (PICO) format was used to search for interventions. The basic combination is P and I; occasionally C is also specified; O is not specified. Search terms are presented in the Appendix A.
4. Search period:
  For all databases, the search period for CQ1 covers all articles included in the databases up to April 30, 2018, and the search period for CQ2 and CQ3 covers all articles included in the databases up to August 10, 2018.
5. Criteria for selection and exclusion of literature:
  The selection criteria for evidence were the following: the target population was adults (aged 18 years or older) with social anxiety disorder, the study design favored RCTs, the comparison groups included a sham group and a waitlist group, the outcome was defined in terms of benefit (improvement in treatment response and social anxiety symptoms) and harm (dropout from treatment due to side effects, adverse events, etc.), and the language of the studies was either English or Japanese.
  A CPG or systematic review paper that met the conditions for adoption is given first priority.
  If there are no CPG or systematic review papers that meet the conditions for adoption, then a de novo systematic review is conducted of individual research papers.
  In the de novo systematic review, all RCTs that meet the recruitment criteria are prioritized.
  If there are no RCTs that meet the recruitment criteria, then observational studies are included.
  If there are no observational studies that meet the recruitment criteria, then the systematic review is not conducted.
6. Methods of evidence evaluation and synthesis:
  The strength evaluation of the total body of evidence is based on methods in the Minds Manual for Clinical Guideline Development 2017. Integration of the total body of evidence is based on qualitative integration, with quantitative integration where appropriate.

### From recommendation decision to finalization and implementation policy

1. Basic policy for making recommendations:
  Decisions on recommendations are based on deliberations of the Preparation Group. If there is no consensus of opinion, then a vote is taken to decide.
  In determining a recommendation and its strength, the “strength of the evidence” and “balance of benefits and harms” that are required in the evaluation and synthesis of evidence and the “diversity of patient values” and “economic perspective” should be considered.
2. Finalization:
  Conduct an external evaluation.
3. Specific methods of external evaluation:
  The external evaluation committee members submit individual comments. The guideline development group discusses the need to change the guideline based on each comment and decides on a response.
4. Schedule for publication:
  The Joint Guideline Development Committee for the Treatment of Anxiety and Obsessive‐Compulsive Disorders makes the final decision on publication. The method of publication is determined in consultation with the guideline development group and Joint Guideline Development Committee for the Treatment of Anxiety and Obsessive‐Compulsive Disorders.

## RECOMMENDATIONS

### CQ1: What is the recommended pharmacotherapy for social anxiety disorder in adults?

#### Background and priority of this issue

Pharmacotherapy has been shown to improve social anxiety symptoms in adults with this condition. However, pharmacotherapy has side effects. It is necessary to identify useful pharmacotherapies that can be used in the current Japanese medical system while considering the benefits and harms.

#### Explanation

##### Summary of evidence: Study designs adopted in the body of evidence

A PubMed search for existing guidelines or systematic reviews of social anxiety disorder in adults revealed that the most recent systematic review on pharmacotherapy was by Williams et al.^1^ Therefore, analyses were conducted with the aim of integrating this systematic review as an existing systematic review with the addition of subsequent literature. The P (Patient/Population) was adults with social anxiety disorder. The I (Intervention) was serotonin 5‐hydroxytryptamine‐1A (5‐HT_1A_) receptor partial agonists, anticonvulsants/γ‐aminobutyric acid (GABA), anticonvulsants/levetiracetam, antipsychotics, benzodiazepines, beta blockers, NaSSAs, SNRIs, SSRIs (fluvoxamine, paroxetine, sertraline, escitalopram, citalopram, and fluoxetine), and others (MAOIs, NARIs, reversible inhibitors of monoamine oxidase A [RIMAs], and serotonin 2 antagonist and reuptake inhibitors [SARIs]). The C (Comparison) was placebo. The O (Outcome) was treatment response (benefit), improvement in social anxiety symptoms (benefit), and dropout from treatment (harm).

The results showed that 26 RCTs of SSRIs and five RCTs of SNRIs had examined the efficacy of pharmacotherapy in adults with social anxiety disorder (see postnote).

Based on benefit‐harm data, high treatment response rates and low dropout rates were important in making these recommendations. SSRIs had a high response rate, with number needed to treat (NNT) = 4.70, and a similar rate of treatment discontinuation as placebo. The response rate for SNRIs was NNT = 4.94, which was similar to SSRIs. The dropout rate was similar to placebo, suggesting a high safety profile. Therefore, we considered SSRIs and SNRIs to be safe and effective therapeutic drugs. However, data on SNRIs were limited relative to venlafaxine.

##### Limitations of research methods (sampling, blinding, allocation concealment, and analytical methods)

Other classes of drugs (antiepileptics and analogs, antipsychotics, benzodiazepines, beta blockers, MAOIs, NARIs, NaSSAs, RIMAs, and SARIs) are not included in the guideline, with or without recommendations, because there have been no new studies after the previous systematic review, and none of them have been adequately studied. For vortioxetine, classified as “other antidepressants,” launched in Japan in November 2019, one RCT of social anxiety disorder was conducted after the previous systematic review, which reported its efficacy and acceptability, but the meta‐analysis was unavailable. Evidence for other classes of drugs is presented in the Appendix S1.

#### Panel meeting

##### What is the quality of the evidence on overall outcomes? (validity of outcomes, consistency of results across studies, directionality of results between studies)

A meta‐analysis of RCTs showed that for reasonable outcomes of benefit (improvements in treatment response and social anxiety symptoms) and harm (dropout from treatment), there was a risk of bias, inconsistency, and other questions for SSRIs and a risk of bias, inconsistency, imprecision, and other questions for SNRIs. Therefore, both classes were downgraded two levels, and the final certainty of evidence was determined to be “low.”

##### What is the balance between benefits and harms? (magnitude of benefit vs. magnitude of harm)

SSRIs and SNRIs produced significant improvements in social anxiety compared with placebo, and dropout rates were similar to placebo. Therefore, the overall benefit (improvements in treatment response and social anxiety symptoms) was determined to be higher than harm (dropout from treatment).

##### What are the patient's values and preferences?

Although side effects of SSRIs and SNRIs were noteworthy, they showed no significant difference in dropout rates from placebo. We concluded that there is no significant uncertainty or diversity in the patients' values and preferences, provided that the effects and side effects are fully explained, and patients consent to the pharmacotherapy of choice.

##### What is the balance between net benefits and costs and resources? (applicability to actual practice)

The cost of treatment is covered by the current national health insurance coverage, with medical remuneration points covering outpatient psychotherapy (540 points for an initial visit of 60 min or more, 330 points for a second visit of less than 30 min, and 400 points for a second visit of 30 min or more) and drug costs (approximately ¥30–¥300 per day, as of April 1, 2019). Medication is available at any psychiatric facility and considered cost‐effective because the treatment has been shown to be effective.

##### Recommendation grading

In the panel meeting discussion, all agreed with the “weak recommendation” label, according to the GRADE assessment.

#### Descriptions of other relevant clinical guidelines

Existing guidelines include the National Institute for Health and Care Excellence (NICE) in the United Kingdom,^2^ S3 in Germany,^3^ and Canadian Clinical Practice Guideline (Canadian CPG) in Canada.^4^ There are no existing guidelines in Japan.

NICE lists escitalopram and sertraline as first‐line pharmacotherapy, and fluvoxamine, paroxetine, and venlafaxine are second‐line drugs due to side effects or discontinuation symptoms, although they are equally effective. Monoamine oxidase inhibitors that are not yet marketed in Japan are considered third‐line drugs due to drug interactions, dietary restrictions, and side effects.

S3 lists escitalopram, paroxetine, sertraline, and venlafaxine as standard drugs for pharmacotherapy. Expert consensus also recommends moclobemide (not available in Japan), a RIMA. Switching to another standard drug if the first standard drug is inadequately effective is also recommended.

The Canadian CPG lists the SSRIs escitalopram, fluvoxamine, paroxetine, and sertraline, the SNRI venlafaxine, and the antiepileptic analog pregabalin as first‐line drugs. As second‐line drugs, it recommends the benzodiazepines alprazolam, bromazepam, and clonazepam, the SSRI citalopram (not yet marketed in Japan), the antiepileptic drug gabapentin, and the MAOI phenelzine (not yet marketed in Japan). Based on the negative evidence, the authors deprecate the beta blockers atenolol and propranolol, the antiepileptic levetiracetam, the antipsychotic quetiapine, and the tricyclic antidepressant imipramine.

#### Treatment monitoring and evaluation

Monitoring and evaluation should be performed by a physician with expertise in the diagnosis and treatment of social anxiety disorder.

#### Potential for future research

There are few studies of pharmacotherapy for inadequate response or intolerance to SSRIs and SNRIs, and RCTs for classes of drugs other than SSRIs and SNRIs are lacking. High‐quality RCTs are desirable in the future.

#### Randomized controlled trial papers covered in this CQ

There are 26 papers on SSRIs and five papers on SNRIs.

Literature used for meta‐analysis of SSRIs:

Allgulander C. Paroxetine in social anxiety disorder: a randomized placebo‐controlled study. *Acta Psychiatr Scand*. 1999;100(3):193–198.
Asakura S, Tajima O, Koyama T. Fluvoxamine treatment of generalized social anxiety disorder in Japan: a randomized double‐blind, placebo‐controlled study. *Int J Neuropsychopharmacol*. 2007;10(2):263–274.
Asakura S, Tsutsui Y, Koyama T. Clinical evaluation of paroxetine hydrochloride hydrate in social anxiety disorder, a double‐blind, placebo‐controlled study. *Jpn J Clin Psychiatry*. 2008;37(6):833–848.
Asakura S, Hayano T, Hagino A, Koyama T. A randomized, double‐blind, placebo‐controlled study of escitalopram in patients with social anxiety disorder in Japan. *Curr Med Res Opin*. 2016;32(4):749–757.
Baldwin D, Bobes J, Stein DJ, Scharwächter I, Faure M. Paroxetine in social phobia/social anxiety disorder: randomized, double‐blind, placebo‐controlled study. Paroxetine Study Group. *Br J Psychiatry*. 1999;175:120–126.
Blomhoff S, Haug TT, Hellström K, Holme I, Humble M, Madsbu HP, et al. Randomized controlled general practice trial of sertraline, exposure therapy and combined treatment in generalized social phobia. *Br J Psychiatry*. 2001;179:23–30.
Book SW, Thomas SE, Randall PK, Randall CL. Paroxetine reduces social anxiety in individuals with a co‐occurring alcohol use disorder. *J Anxiety Disord*. 2008;22(2):310–318.
Davidson J, Yaryura‐Tobias J, DuPont R, Stallings L, Barbato LM, van der Hoop RG, et al. Fluvoxamine‐controlled release formulation for the treatment of generalized social anxiety disorder. *J Clin Psychopharmacol*. 2004;24(2):118–125.
Davidson JR, Foa EB, Huppert JD, Keefe FJ, Franklin ME, Compton JS, et al. Fluoxetine, comprehensive cognitive behavioral therapy, and placebo in generalized social phobia. *Arch Gen Psychiatry*. 2004;61(10):1005–1013.
Furmark T, Appel L, Michelgård A, Wahlstedt K, Ahs F, Zancan S, et al. Cerebral blood flow changes after treatment of social phobia with the neurokinin‐1 antagonist GR205171, citalopram, or placebo. *Biol Psychiatry*. 2005;58(2):132–142.
Kasper S, Stein DJ, Loft H, Nil R. Escitalopram in the treatment of social anxiety disorder: randomized, placebo‐controlled, flexible‐dosage study. *Br J Psychiatry*. 2005;186:222–226.
Kobak KA, Greist JH, Jefferson JW, Katzelnick DJ. Fluoxetine in social phobia: a double‐blind, placebo‐controlled pilot study. *J Clin Psychopharmacol*. 2002;22(3):257–262.
Lader M, Stender K, Bürger V, Nil R. Efficacy and tolerability of escitalopram in 12‐ and 24‐week treatment of social anxiety disorder: randomized, double‐blind, placebo‐controlled, fixed‐dose study. *Depress Anxiety*. 2004;19(4):241–248.
Lepola U, Bergtholdt B, St Lambert J, Davy KL, Ruggiero L. Controlled‐release paroxetine in the treatment of patients with social anxiety disorder. *J Clin Psychiatry*. 2004;65(2):222–229.
Liebowitz MR, Stein MB, Tancer M, Carpenter D, Oakes R, Pitts CD. A randomized, double‐blind, fixed‐dose comparison of paroxetine and placebo in the treatment of generalized social anxiety disorder. *J Clin Psychiatry*. 2002;63(1):66–74.
Liebowitz MR, DeMartinis NA, Weihs K, Londborg PD, Smith WT, Chung H, et al. Efficacy of sertraline in severe generalized social anxiety disorder: results of a double‐blind, placebo‐controlled study. *J Clin Psychiatry*. 2003;64(7):785–792.
Liebowitz MR, Gelenberg AJ, Munjack D. Venlafaxine extended release vs placebo and paroxetine in social anxiety disorder. Arch Gen Psychiatry. 2005;62(2):190–198.
NCT00318669. Social Anxiety Disorder Study Of Paroxetine. Available from: https://clinicaltrials.gov/ct2/show/NCT00318669
NCT00397722. Treatment Of Patients With Social Anxiety Disorder. Available from: https://clinicaltrials.gov/ct2/show/study/NCT00397722
NCT00403962. A Combination Therapy In Patients With Social Anxiety Disorder. Available from: https://clinicaltrials.gov/ct2/show/NCT00403962
Nordahl HM, Vogel PA, Morken G, Stiles TC, Sandvik P, Wells A. Paroxetine, cognitive therapy or their combination in the treatment of social anxiety disorder with and without avoidant personality disorder: a randomized clinical trial. *Psychother Psychosom*. 2016;85(6):346–356.
Randall CL, Johnson MR, Thevos AK, Sonne SC, Thomas SE, Willard SL, et al. Paroxetine for social anxiety and alcohol use in dual‐diagnosed patients. *Depress Anxiety*. 2001;14(4):255–262.
Stein MB, Liebowitz MR, Lydiard RB, Pitts CD, Bushnell W, Gergel I. Paroxetine treatment of generalized social phobia (social anxiety disorder): a randomized controlled trial. *JAMA*. 1998;280(8):708–713.
Stein MB, Fyer AJ, Davidson JR, Pollack MH, Wiita B. Fluvoxamine treatment of social phobia (social anxiety disorder): a double‐blind, placebo‐controlled study. *Am J Psychiatry*. 1999;156(5):756–760.
Van Ameringen MA, Lane RM, Walker JR, Bowen RC, Chokka PR, Goldner EM, et al. Sertraline treatment of generalized social phobia: a 20‐week, double‐blind, placebo‐controlled study. *Am J Psychiatry*. 2001;158(2):275–281.
Westenberg HG, Stein DJ, Yang H, Li D, Barbato LM. A double‐blind placebo‐controlled study of controlled release fluvoxamine for the treatment of generalized social anxiety disorder. *J Clin Psychopharmacol*. 2004;24(1):49–55.

Literature used for meta‐analysis of SNRIs:

Liebowitz MR, Mangano RM, Bradwejn J, Asnis G. A randomized controlled trial of venlafaxine extended release in generalized social anxiety disorder. *J Clin Psychiatry*. 2005;66(2):238–247.
Liebowitz MR, Gelenberg AJ, Munjack D. Venlafaxine extended release vs placebo and paroxetine in social anxiety disorder. *Arch Gen Psychiatry*. 2005;62(2):190–198.
NCT01316302. 12‐Week Study of Pristiq (Desvenlafaxine) Social Anxiety Disorder. Available from: https://clinicaltrials.gov/ct2/show/NCT01316302
Rickels K, Mangano R, Khan A. A double‐blind, placebo‐controlled study of a flexible dose of venlafaxine ER in adult outpatients with generalized social anxiety disorder. *J Clin Psychopharmacol*. 2004;24(5):488–496.
Stein MB, Pollack MH, Bystritsky A, Kelsey JE, Mangano RM. Efficacy of low and higher dose extended‐release venlafaxine in generalized social anxiety disorder: a 6‐month randomized controlled trial. *Psychopharmacology (Berl)*. 2005;177(3):280–288.

REFERENCES FOR CQ11

Williams
T
, 
Hattingh
CJ
, 
Kariuki
CM
, 
Tromp
SA
, 
van Balkom
AJ
, 
Ipser
JC
, et al. Pharmacotherapy for social anxiety disorder (SAnD). Cochrane Database Syst Rev. 2017;10(10):Cd001206.2904873910.1002/14651858.CD001206.pub3PMC63609272
National Institute for Health and Care Excellence (NICE)
. Social Anxiety Disorder: Recognition, Assessment and Treatment (Clinical Guideline [CG159]). Leicester: British Psychological Society; 2013. Available from: http://guidance.nice.org.uk/CG159
318690483

Bandelow
B
, 
Lichte
T
, 
Rudolf
S
, 
Wiltink
J
, 
Beutel
ME
. The diagnosis of and treatment recommendations for anxiety disorders. Dtsch Arztebl Int. 2014;111(27–28):473–480.2513872510.3238/arztebl.2014.0473PMC41874074

Katzman
MA
, 
Bleau
P
, 
Blier
P
, 
Chokka
P
, 
Kjernisted
K
, 
Van Ameringen
M
, et al. Canadian clinical practice guidelines for the management of anxiety, posttraumatic stress and obsessive‐compulsive disorders. BMC Psychiatry. 2014;14(Suppl 1):S1.2508158010.1186/1471-244X-14-S1-S1PMC4120194

### CQ2: What is the recommended psychotherapy (psychological intervention) for social anxiety disorder in adults?

#### Cognitive behavioral therapy (CBT)

CBT, which was developed specifically for the treatment of social anxiety disorder, should be structured with a total of approximately 14 sessions that are performed over approximately 4 months so each individual session lasts approximately 60–90 min. Group therapy should consist of one 120‐ to 150‐min group session (two to three patients per therapist), for a total of approximately 12 sessions that are conducted over approximately 3 months.

Treatment should include the following:

(Clark & Wells model)

- Psychoeducation on social anxiety.
- Experiential exercises that demonstrate negative effects of self‐attention and safety‐seeking behavior.
- Video feedback to correct distorted negative self‐image.
- Systematic training to pay attention to external stimuli.
- In‐session behavioral experiments that examine negative beliefs and homework related to them.
- Discrimination training and rewriting to address traumatic memories of distressing social situations.
- Review and modification of core beliefs.
- Modification of anticipatory anxiety and ruminations related to social situations.
- Relapse prevention.

(Heimberg model)

- Psychoeducation on social anxiety.
- Cognitive restructuring.
- Gradual exposure to feared social situations (both in‐session and as homework).
- Review and modification of core beliefs.
- Relapse prevention.

#### Self‐help with support based on CBT

Supported self‐help for social anxiety disorder consists of the following:

- Typically, approximately nine sessions are conducted over 3–4 months using self‐help materials based on CBT with support.
- The use of self‐help materials is supported by a therapist via face‐to‐face meetings or telephone for a total of approximately 3 h over the course of a treatment series.

#### Background and priority of this issue

Social anxiety disorder is a common disorder in Japan, with a reported 12‐month prevalence of 0.8%. Considering that the median age of onset in the United States is 13 years and that symptoms persist thereafter, the impact on daily life and quality of life is critical. Therefore, identifying the recommended psychotherapy is a priority.

Psychotherapy has been shown to improve social anxiety symptoms. In addition to CBT, various other types of psychotherapy are used, including mindfulness cognitive therapy, acceptance and commitment therapy (third‐generation behavior therapy), psychodynamic therapy, and Morita therapy. Additionally, guidelines are needed for recommended forms of psychotherapy, such as individual or group sessions, and self‐help. Therefore, it is necessary to identify useful psychotherapies that can be provided in the current Japanese medical system.

#### Explanation

##### Summary of evidence

We first searched the NGC and IGL for existing clinical guidelines on social anxiety disorder in adults. The NICE guideline^1^ was identified as specific to social anxiety disorder. The P (Patient/Population) was identified as adults with social anxiety disorder. The I (Intervention) was CBT, interpersonal therapy, psychodynamic therapy (psychoanalytic psychotherapy), Morita therapy, and others. The C (Comparison) was waiting, placebo, and other psychosocial interventions. The O (Outcome) was treatment response (benefit), improvement in social anxiety symptoms (benefit), and dropout from treatment (harm). Next, the possibility of using the NICE guideline in this CQ was discussed with reference to the “Policy for Using Existing Systematic Reviews” in the Minds Manual for Clinical Guideline Development 2017. We first evaluated the NICE guideline using the Appraisal of Guidelines for Research and Evaluation II (AGREE II). We then confirmed that the NICE guideline satisfied the PIC (from PICO) of this CQ. Note that the O in this CQ included “dropout” and “improvement in social anxiety symptoms” but not “treatment responsiveness.” However, because “recovery” was included in the outcome of this CQ as an umbrella concept for “treatment responsiveness,” we determined that all PICOs were met (a systematic review using “responsiveness” as the outcome is required in the future). After the NICE guideline was published in 2013, the guideline included evidence of 4 years of follow‐up in Surveillance Report 2017.^2^ Based on the above, we decided to use the NICE guideline under the condition that the evidence should be updated following the publication of Surveillance Report 2017. Specifically, we first searched for studies that were published after November 27, 2016, using the same literature search strategy as the NICE guideline and identified studies that met the PICO of this CQ. To extract Japanese‐language literature, we also searched the Ichu‐shi Web using the same keywords (without specifying the time period). Next, data extraction and bias risk assessment were performed for each identified study. Finally, we prepared a descriptive summary of each study. Note that we combined the results of the studies that were identified in this systematic review with those that were included in the network meta‐analysis of the NICE guideline and did not re‐analyze or re‐evaluate the total body of results.

##### Literature search and screening results

After searching databases and conducting primary and secondary screening, we identified 11 studies that met this CQ. These included CBT‐based intervention versus treatment waitlist (*n* = 7), psychodynamic therapy versus treatment waitlist (*n* = 1), and other psychotherapy versus treatment waitlist/control (*n* = 3).

We tracked studies that were published after the NICE guideline and Surveillance Report 2017 (after November 27, 2016) and found no evidence that conflicted with results and recommendations of the NICE guideline and Surveillance Report 2017. Based on the above, we developed recommendations for this CQ based on recommendations for psychotherapy in the NICE guideline and Surveillance Report 2017.

A paper^3^ that presented the results of the network meta‐analysis of the NICE guideline found that psychotherapy (CBT, self‐help, exposure therapy/social skills training, and short‐term forced psychodynamic therapy) contributed to the improvement in social anxiety symptoms. Of these, individual CBT was the most effective and the only one of the various treatments, including pharmacotherapy, that outperformed both the waitlist and placebo groups. Although dropout from treatment has not been evaluated overall because of the paucity of reported studies, the network meta‐analysis that is described above is based on the principle of intention‐to‐treat, which takes dropout cases into account.

#### Panel meeting

##### What is the quality of evidence on overall outcomes?

For this comparison that is included in the NICE guideline, a GRADE assessment could not be made because the required data for individual studies were unavailable. The network meta‐analysis as a whole had no serious problems with non‐directness, inconsistency, or imprecision, but there were some minor bias risks and some serious issues with publication bias. Therefore, together, they were downgraded by two levels, and the final certainty of evidence was determined to be “low.”

The results of the NICE guideline systematic review and meta‐analysis on which this recommendation was based were mostly from Western countries, with minimal data from Asian countries. However, because there are findings that outcomes of clinical trials on psychotherapy for social anxiety disorder do not vary significantly across cultures and that minor modifications that consider cultural differences can be expected to be effective,^4^, ^5^ the conclusion of this recommendation is unchanged.

##### What is the balance between benefits and harms?

Individual CBT has produced significant improvements in social anxiety symptoms. Although dropout rates are not addressed in the network meta‐analysis, there is no clear evidence from existing research that CBT increases dropout rates. Therefore, the overall benefit (improvement in social anxiety symptoms) was determined to be higher than harm (dropout from treatment).

##### What are the patient's values and preferences?

In this study, we were unable to search for articles that systematically investigated values and preferences of Japanese patients with social anxiety disorder. However, there have been surveys on psychiatric disorders and anxiety disorders in other countries.^6^, ^7^, ^8^, ^9^, ^10^ Overall, patients prefer psychotherapy over pharmacotherapy.

Based on the above, we propose the psychotherapy that is described above because it is assumed to be preferred by patients and because there are fewer side effects than would occur with pharmacotherapy. We propose individual CBT, which is the most clinically effective and cost‐effective among various treatments for social anxiety disorder. However, it is necessary to carefully consider the patient's preferences after fully explaining that psychotherapy generally requires more frequent visits and longer sessions than pharmacotherapy and that the patient may need to go to a medical institution that can provide a specific type of psychotherapy and may need to bear the costs.

##### What is the balance between benefits and costs and resources?

Although treatment costs can be estimated, and the degree of certainty is relatively high, calculation requirements and medical remuneration points fluctuate with revisions to medical fees.

High‐frequency individual CBT (e.g., 16 sessions of weekly CBT) is expected to cost more than pharmacotherapy in the short term. However, as shown by the NICE guideline results, it is still cost‐effective. Even when modeled for Japan, the costs are likely to be lower than the threshold of willingness to pay (incremental cost‐effectiveness ratio = ¥5 million), which is the average amount of money that the Japanese population is willing to pay for treatment. A health economic analysis based on a Japanese clinical trial (see Appendix S1 of Yoshinaga et al.^11^) showed that the cost‐effectiveness of individual CBT was significantly lower than the upper limit of average willingness to pay in the Japanese population. However, from the perspective of a medical facility, the maximum revenue from national health insurance (medical remuneration points) per an individual session of approximately 60–90 min is expected to be 480 points, whereas the salary and other costs of a specialist for 60–90 min would often exceed that amount. This may be a barrier to the widespread use of CBT in insured practices.

If the supply of individual CBT under national health insurance is insufficient because of the above reasons, among others, some patients may be unable to receive psychotherapy unless they pay for it out‐of‐pocket, which raises the issue of fairness because the out‐of‐pocket costs will be higher than for insured treatment. Additionally, transportation costs may also be incurred if the medical institution where a particular psychotherapy is provided is not nearby and the patient must travel to a distant location.

Although interventions require specific medical expenses, as mentioned above, some effectiveness can be expected. Additionally, medical expenses are covered by national health insurance, and independent‐living medical care for people with disabilities may be available in some cases. A cost‐effectiveness analysis that was conducted by the NICE guideline found individual CBT was the most cost‐effective, which is thought to be attributable to strong therapeutic effectiveness and the fact that effectiveness is sustained over time.

##### Recommendation grading

In the panel meeting discussion, all members unanimously agreed to “suggest individual CBT” and “suggest self‐help support based on CBT with conditions” because of the lack of a GRADE evaluation and the low overall certainty of evidence.

#### Descriptions of other relevant clinical guidelines

There are no other treatment guidelines for social anxiety disorder in Japan. Internationally, there is the NICE guideline from the UK that we use here. The conclusions of the NICE guideline are the same as those for the present guideline.

#### Treatment monitoring and evaluation

Treatment monitoring, evaluation, follow‐up, and support for the patient are provided by the therapist. All interventions should be provided by therapists with sufficient knowledge and skills (competencies). For psychotherapy, this should be based on treatment manuals that include guidance on the structure and duration of interventions. Therapists should consider the competency framework (e.g., standards of training) that are set out in relevant treatment manuals and should also consider the following points:

- Receiving regular access to quality supervision (guidance from a skilled practitioner) based on information about treatment outcomes.
- Using outcome measures (e.g., Liebowitz Social Anxiety Scale [LSAS] or Social Phobia Inventory [SPIN]) at every session and allowing patients with social anxiety disorder to be involved in reflecting on treatment effects.
- Monitoring and evaluating treatment adherence and therapist competency, such as using video/audio recordings and, where appropriate, external audit and scrutiny.

#### Potential for future research

- There is a need for reanalysis/update of the NICE guideline to include evidence from the network meta‐analysis. Systematic reviews/meta‐analyses of adverse effects of psychotherapy are also needed.
- Recently, evidence for cross‐diagnostic interventions (e.g., unified protocol) for anxiety disorders, including social anxiety disorder, has also been published. Therefore, there is a need to consider whether social anxiety disorder‐specific or cross‐diagnostic interventions are more effective.

#### Randomized controlled trial papers covered in this CQ

This CQ is based on the network meta‐analysis that was conducted under the following NICE guideline:

National Institute for Health and Care Excellence (NICE). *Social Anxiety Disorder: Recognition, Assessment and Treatment (Clinical Guideline [CG159])*. Leicester: British Psychological Society; 2013. Available from: http://guidance.nice.org.uk/CG159

Additionally, a literature search and Japanese literature search were conducted after Surveillance Report 2017, and the studies that are included in the evidence update are the following:

Barlow DH, Farchione TJ, Bullis JR, Gallagher MW, Murray‐Latin H, Sauer‐Zavala S, et al. The unified protocol for transdiagnostic treatment of emotional disorders compared with diagnosis‐specific protocols for anxiety disorders: a randomized clinical trial. *JAMA Psychiatry*. 2017;74(9):875–884.
Bouchard S, Dumoulin S, Robillard G, Guitard T, Klinger É, Forget H, et al. Virtual reality compared with in vivo exposure in the treatment of social anxiety disorder: a three‐arm randomized controlled trial. *Br J Psychiatry*. 2017;210(4):276–283.
Craske MG, Niles AN, Burklund LJ, Wolitzky‐Taylor KB, Vilardaga JC, Arch JJ, et al. Randomized controlled trial of cognitive behavioral therapy and acceptance and commitment therapy for social phobia: outcomes and moderators. *J Consult Clin Psychol*. 2014;82(6):1034–1048.
Ivanova E, Lindner P, Ly KH, Dahlin M, Vernmark K, Andersson G, et al. Guided and unguided Acceptance and Commitment Therapy for social anxiety disorder and/or panic disorder provided via the Internet and a smartphone application: a randomized controlled trial. *J Anxiety Disord*. 2016;44:27–35.
Jazaieri H, Goldin PR, Gross JJ. Treating social anxiety disorder with CBT: impact on emotion regulation and satisfaction with life. *Cogn Ther Res*. 2017;41(3):406–416.
Johansson R, Hesslow T, Ljótsson B, Jansson A, Jonsson L, Färdig S, et al. Internet‐based affect‐focused psychodynamic therapy for social anxiety disorder: a randomized controlled trial with 2‐year follow‐up. *Psychotherapy*. 2017;54(4):351–360.
Lazarov A, Pine DS, Bar‐Haim Y. Gaze‐contingent music reward therapy for social anxiety disorder: a randomized controlled trial. *Am J Psychiatry*. 2017;174(7):649–656.
LeBouthillier DM, Asmundson GJG. The efficacy of aerobic exercise and resistance training as transdiagnostic interventions for anxiety‐related disorders and constructs: A randomized controlled trial. *J Anxiety Disord*. 2017;52:43–52.
Naim R, Kivity Y, Bar‐Haim Y, Huppert JD. Attention and interpretation bias modification treatment for social anxiety disorder: a randomized clinical trial of efficacy and synergy. *J Behav Ther Exp Psychiatry*. 2018;59:19–30.
Riccardi CJ, Korte KJ, Schmidt NB. False safety behavior elimination therapy: a randomized study of a brief individual transdiagnostic treatment for anxiety disorders. *J Anxiety Disord*. 2017;46:35–45.
Stolz T, Schulz A, Krieger T, Vincent A, Urech A, Moser C, et al. A mobile app for social anxiety disorder: a three‐arm randomized controlled trial comparing mobile and PC‐based guided self‐help interventions. *J Consult Clin Psychol*. 2018;86(6):493–504.

REFERENCES FOR CQ21
National Institute for Health and Care Excellence (NICE)
. Social Anxiety Disorder: Recognition, Assessment and Treatment (Clinical Guideline [CG159]). Leicester: British Psychological Society; 2013. Available from: http://guidance.nice.org.uk/CG159
318690482
National Institute for Health and Care Excellence (NICE). Surveillance report 2017 – Social Anxiety Disorder: Recognition, Assessment and Treatment
. NICE guideline CG159. Leicester: British Psychological Society; 2013. Available from: https://www.nice.org.uk/guidance/cg159/resources/surveillance‐report‐2017‐social‐anxiety‐disorder‐recognition‐assessment‐and‐treatment‐2013‐nice‐guideline‐cg159‐4484818333/chapter/Surveillance‐decision?tab=evidence
318690483

Mayo‐Wilson
E
, 
Dias
S
, 
Mavranezouli
I
, 
Kew
K
, 
Clark
DM
, 
Ades
AE
, et al. Psychological and pharmacological interventions for social anxiety disorder in adults: a systematic review and network meta‐analysis. Lancet Psychiatry. 2014;1(5):368–376.2636100010.1016/S2215-0366(14)70329-3PMC42878624

Hernandez Hernandez
ME
, 
Waller
G
, 
Hardy
G
. Cultural adaptations of cognitive behavioural therapy for Latin American patients: unexpected findings from a systematic review. Cogn Behav Therapist. 2020;13:e57.5

Jankowska
M
. Cultural modifications of cognitive behavioural treatment of social anxiety among culturally diverse clients: a systematic literature review. Cogn Behav Therapist. 2019;12:e7.6

Backenstrass
M
, 
Joest
K
, 
Frank
A
, 
Hingmann
S
, 
Mundt
C
, 
Kronmüller
KT
. Preferences for treatment in primary care: a comparison of nondepressive, subsyndromal and major depressive patients. Gen Hosp Psychiatry.
2006;28(2):178–180.1651607010.1016/j.genhosppsych.2005.10.0017

Deacon
BJ
, 
Abramowitz
JS
. Patients' perceptions of pharmacological and cognitive‐behavioral treatments for anxiety disorders. Behav Therapy. 2005;36(2):139–145.10.1016/j.brat.2006.04.010167847228

McHugh
RK
, 
Whitton
SW
, 
Peckham
AD
, 
Welge
JA
, 
Otto
MW
. Patient preference for psychological vs pharmacologic treatment of psychiatric disorders: a meta‐analytic review. J Clin Psychiatry. 2013;74(6):595–602.2384201110.4088/JCP.12r07757PMC41561379

Ogrodniczuk
JS
, 
Piper
WE
, 
Joyce
AS
, 
Abbass
AA
. Alexithymia and treatment preferences among psychiatric outpatients. Psychother Psychosom.
2009;78(6):383–384.1973840710.1159/00023598110

Zafar
AM
, 
Jawaid
A
, 
Ashraf
H
, 
Fatima
A
, 
Anjum
R
, 
Qureshi
SU
. Psychotherapy as a treatment modality for psychiatric disorders: perceptions of general public of Karachi, Pakistan. BMC Psychiatry. 2009;9:37.1952750610.1186/1471-244X-9-37PMC270237611

Yoshinaga
N
, 
Kubota
K
, 
Yoshimura
K
, 
Takanashi
R
, 
Ishida
Y
, 
Iyo
M
, et al. Long‐term effectiveness of cognitive therapy for refractory social anxiety disorder: one‐year follow‐up of a randomized controlled trial. Psychother Psychosom. 2019;88(4):244–246.3112159210.1159/000500108

### CQ3: What are the recommendations regarding monotherapy and combination therapy for social anxiety disorder in adults in terms of pharmacotherapy and psychotherapy (psychological interventions)?

Recommendation:

There is no recommendation for the use of combined pharmacotherapy and psychotherapy (psychological intervention) for social anxiety disorder in adults.

#### Background and priority of this issue

In Japan, social anxiety disorder is a common disorder with a reported 12‐month prevalence of 0.8%. Considering that the median age of onset in the US is 13 years and that symptoms have usually persisted since adolescence, the impact on daily life and quality of life is significant, and identifying recommended treatments is a priority. Medication and psychotherapy (psychological intervention) are the most common treatment options, but it is also necessary to clarify whether they should be used in combination.

#### Explanation

##### Summary of evidence

We first searched the NGC and IGL for existing clinical guidelines on social anxiety disorder in adults. The NICE guideline^1^ was identified as specific to social anxiety disorder. The P (Patient/Population) was adult social anxiety disorder. The I (Intervention) was 5‐HT_1A_ receptor partial agonists, anticonvulsants/GABAs, anticonvulsants/levetiracetam, antipsychotics, benzodiazepines, beta blockers, NaSSAs, SNRIs, SSRIs (fluvoxamine, paroxetine, sertraline, escitalopram, citalopram, and fluoxetine), MAOIs, NARIs, RIMAs, SARIs, CBT, interpersonal therapy, psychodynamic therapy (psychoanalytic therapy), Morita therapy, and others. The C (Comparison) was waiting, placebo, or combination treatments (pharmacotherapy/psychotherapy). The O (Outcome) was treatment response (benefit), improvement in social anxiety symptoms (benefit), and dropout from treatment (harm). Next, the possibility of using the NICE guideline in this CQ was discussed with reference to the “Policy for Using Existing Systematic Reviews” in the Minds Manual for Clinical Guideline Development Manual 2017. First, we evaluated the NICE guideline using the AGREE II. Second, we confirmed that the NICE guideline satisfied the PIC (from PICO) of this CQ. Note that the O of this CQ included “dropout” and “improvement in social anxiety symptoms” but not “treatment responsiveness.” However, because “recovery” was included as an umbrella concept for “treatment responsiveness,” we determined that all PICOs were met (a systematic review using “responsiveness” as the outcome is required in the future). Third, 4 years after the NICE guideline was published in 2013, the guideline included follow‐up evidence in Surveillance Report 2017.^2^ Based on the above, we decided to use the NICE guideline on the condition that the evidence be updated following the publication of Surveillance Report 2017. Specifically, we searched for studies that were published after November 27, 2016, using the same literature search strategy as the NICE guideline and reviewed those that met the PICO for this CQ. To extract Japanese‐language literature, we also searched the JAMA Web using the same keywords (without specifying the time period).

##### Literature search and screening results

After database searches and primary and secondary screening, no studies that met this CQ were identified. Based on these results, we decided to follow the NICE guideline for evidence regarding CQ3 recommendations. Studies of combination therapy that were included in the network meta‐analysis of the NICE guideline were all different combinations (one study each), meaning that certainty was very low and the balance between desirable and undesirable effects could not be determined.

#### Panel meeting

##### What is the quality of the evidence on overall outcomes?

A GRADE assessment could not be made for this comparison that is included in the NICE guideline because the required data for individual studies were unavailable. Studies of combination therapies that were included in the network meta‐analysis of the NICE guideline were all regarding different combinations, and overall certainty of the evidence was very low.

##### What is the balance between benefits and harms?

Studies of combination therapies that were included in the network meta‐analysis of the NICE guideline were all regarding different combinations (one study each). Therefore, we were unable to determine the balance between desirable and undesirable effects.

##### What are the patient's values and preferences?

There are patients who do not wish to receive pharmacotherapy or psychotherapy. Combination therapy may also create additional risks and burdens for patients (e.g., side effects of additional SSRIs, hospital visits and costs when there is no nearby medical facility that can provide CBT). It is unclear whether the benefits of combination therapy outweigh these risks and burdens, and there is no clear recommendation for combination therapy. If a patient requests such therapy, then benefits and harms of these therapies should be fully explained to the patient, and the therapies/proposals should be tailored to the patient's preferences.

##### What is the balance between net benefits and costs and resources?

Cognitive behavioral therapy costs 480 medical remuneration points if it is performed by a physician and 350 points if it is performed jointly by a physician and nurse practitioner. Cognitive behavioral therapy requires an interview that lasts at least 30 min and can only be billed to insurance for 16 weeks. Essentially, it is conducted once weekly for 50 min for 12–18 sessions. When pharmacotherapy is used, it is exclusively billed in conjunction with outpatient psychotherapy. This is covered by national health insurance at 400 medical remuneration points for psychotherapy that lasts 30 min or more and 330 points for psychotherapy that lasts <30 min, plus a prescription fee of 42 points (with reductions that depend on the type of drug that is administered and its duration) and a prescription fee of 68 points (with reductions that depend on the type of drug that is administered and its duration). All medical remuneration points that are listed here are as of August 16, 2019. However, because CBT by psychologists cannot be billed to insurance, only a few treatment facilities provide CBT that can be billed to insurance, and many other medical facilities provide it without insurance coverage. Therefore, the cost of CBT may be higher. Nonetheless, it is difficult to compare the total cost of CBT with long‐term pharmacotherapy because of its limited treatment period. However, evidence of whether benefits of using both therapies in combination outweigh costs of treatment, which are certain to be higher than costs of a single treatment alone, is inconclusive. Additionally, pharmacotherapy can be administered at many medical institutions, but CBT can only be provided at a limited number of institutions, which is a significant imbalance in terms of resources.

##### Recommendation grading

In the panel meeting discussion, all agreed that there were no recommendations about whether or not to implement combined pharmacotherapy and psychotherapy because of the lack of GRADE evaluation and very low overall certainty of the evidence.

#### Descriptions of other relevant clinical guidelines

No other treatment guidelines for social anxiety disorder exist in Japan. Internationally, there is the UK NICE guideline (2013) that we use here. The conclusions in both are the same.

#### Treatment monitoring and evaluation

Treatment monitoring, evaluation, follow‐up, and support for the patient are provided by the therapist. Treatment responsiveness and the degree of improvement in social anxiety symptoms should be used as measures of efficacy. Improvement in social anxiety symptoms may also be monitored by the LSAS. The occurrence of side effects that lead to treatment dropout and evaluation of treatment adherence are also important indicators of evaluation in using this guideline. In the case of CBT, this should be done with supervision.

#### Potential for future research

- Because of the lack of evidence for this CQ, the network meta‐analysis needs to be reanalyzed and updated to include evidence from the time that the network meta‐analysis for the NICE guideline was conducted.
- Comparative studies of treatment response, remission rates, and dropout rates for the three groups of CBT, SSRIs, and CBT plus SSRIs are needed.
- Studies of treatment response, remission rates, and dropout rates for additional SSRIs in the group of partial responders to CBT are needed.
- Studies of treatment response, remission rates, and dropout rates with the addition of CBT to partial responders to SSRIs are needed.

#### Randomized controlled trial papers covered in this CQ

This CQ is based on the network meta‐analysis that was conducted under the following NICE guideline:

National Institute for Health and Care Excellence (NICE). *Social Anxiety Disorder: Recognition, Assessment and Treatment (Clinical Guideline [CG159])*. Leicester: British Psychological Society; 2013. Available from: http://guidance.nice.org.uk/CG159

Additionally, a literature search after Surveillance Report 2017 and a Japanese literature search were performed, but no studies were identified that matched this CQ.

REFERENCES FOR CQ31
National Institute for Health and Care Excellence (NICE)
. Social Anxiety Disorder: Recognition, Assessment and Treatment (Clinical Guideline [CG159]). Leicester: British Psychological Society; 2013. Available from: http://guidance.nice.org.uk/CG159
318690482
National Institute for Health and Care Excellence (NICE). Surveillance report 2017 – Social Anxiety Disorder: Recognition, Assessment and Treatment (2013)
. NICE Guideline CG159. Leicester: British Psychological Society; 2017 Available from: https://www.nice.org.uk/guidance/cg159/resources/surveillance‐report‐2017‐social‐anxiety‐disorder‐recognition‐assessment‐and‐treatment‐2013‐nice‐guideline‐cg159‐4484818333/chapter/Surveillance‐decision?tab=evidence
31869048

## POST‐PUBLICATION INITIATIVES

### Post‐publication organizational structure

After release of the guideline, the Japanese Society of Anxiety and Related Disorders and Japanese Society of Neuropsychopharmacology will continue the Joint Clinical Practice Guideline Development Committee for Anxiety and Obsessive‐Compulsive Disorders. The Guideline Development Office will manage the committee activities.

### Introduction: Factors that promote and hinder application of the guideline

The guideline has been registered on the Minds Guideline Library website and made available as a downloadable PDF file from the websites of the Japanese Society of Anxiety and Related Disorders and Japanese Society of Neuropsychopharmacology so that it can be widely accessed by medical specialists, non‐specialists, patients, and their families. An abridged version of the guideline, educational materials, and a book with detailed explanations will also be prepared to disseminate the guideline.

Factors that promote and hinder application of the guideline, based on feedback from stakeholders, are described below. Factors that promote pharmacotherapy include the fact that SSRIs are covered by national health insurance, and generic drugs also exist and are easy to use. A hindering factor is the fact that benzodiazepines are more likely to be used clinically, which may disincentivize the use of SSRIs. One factor that promotes psychotherapy is that CBT, when conducted by physicians, is also covered by national health insurance. Conversely, factors that hinder psychotherapy include the lack of sufficient medical remuneration points for CBT and the lack of insurance coverage when it is conducted by a certified public psychologist. Although the above factors did not influence guideline development or recommendations, it is necessary to investigate and research in the future the influence of factors that promote and hinder adherence to guideline recommendations in actual clinical practice.

### Effectiveness evaluation: Criteria for monitoring (auditing)

We plan to conduct research to examine clinical effects of introducing the guideline using quality indicators and other methods. For members of the Japanese Society of Anxiety and Related Disorders and Japanese Society of Neuropsychopharmacology, a study will be conducted to monitor (audit) and report annually (frequency and interval of measurement) the number of patients with social anxiety disorder aged 18 years and older (adults) and the number of cases in which the guideline recommendations were applied (evaluation of compliance) and symptom improvement or treatment dropout as measured by the LSAS at 0, 8, and 16 weeks (to assess the impact of implementation) by individual physicians and medical institutions to determine the extent to which the guideline recommendations are being applied.

### Revisions

Every 3 years, or when otherwise necessary (e.g., when a new treatment is introduced), revision of the guideline will be considered. The Guideline Development Office will convene the Guideline Development Committee to revise the guideline.

## AUTHOR CONTRIBUTIONS

Conception and design: All authors. Acquisition and analysis of data: NY, HY, YF, NM, and YK. Interpretation of data: All authors. Project management: SA, TI, and ES. Drafting the manuscript: SA, NY, HY, YF, NM, and YK. Revising the manuscript for intellectual content: All authors. Final approval of the manuscript: All authors.

## FUNDING INFORMATION

This guideline was developed by the Japanese Society of Anxiety and Related Disorders and Japanese Society of Neuropsychopharmacology, and both societies provided funding for development of the guideline. See “Funding” section in main text for more detailed information.

## CONFLICT OF INTEREST STATEMENT

Takeshi Inoue has received lecture fees (Kyowa Pharmaceutical, Pfizer, Mitsubishi Tanabe Pharma, Otsuka Pharmaceutical, and MSD), and endowments (Eisai, Sumitomo Dainippon Pharma, Mitsubishi Tanabe Pharma, MSD, Pfizer, and Meiji Seika Pharma). Yoshihiro Kanai has received lecture fees (Advantage Risk Management Co., Ltd.). Eiji Shimizu has received research funding (Sumitomo Dainippon Pharma). Satoshi Asakura has received lecture fees (Mitsubishi Tanabe Pharma Corporation). See “Conflicts of interest (for the past 3 years)” section in main text for more detailed information.

## ETHICS STATEMENT

N/A.

## Supporting information


Appendix S1

## Data Availability

Supporting data for this guideline and recommendations are available from the Minds Guideline Library website at https://minds.jcqhc.or.jp/n/med/4/med0458/G0001312.

## APPENDIX A METHODS FOR EVIDENCE SEARCH

Below is a list of search terms and search formulas used for some databases.

### CQ1: What is the recommended pharmacotherapy for social anxiety disorder in adults?

Title: Search for SR/MA articles and individual research articles on pharmacotherapy for social anxiety disorder in adults.

**Table:**

Database	PubMed
Date	Sep 20, 2018
Searcher	NM/YF

Database search results.

**Table:**

#	Search formula	Number of hits
1	All Fields: social phobia OR social anxiety disorder	15 446
2	All Fields: medication OR pharmacotherapy OR treatment	10 562 918
3	1 AND 2	8022
4	TX: taijin kyofusho OR taijinkyofu	28
5	TX: social anxiety OR social phobia	12 829
6	3 OR 4 OR 5	9685
7	limit 6 to year = “2017/08/01–Apr 2018/04/30”	547

### CQ2: What is the recommended psychotherapy for social anxiety disorder in adults?

Title: Search for SR/MA articles and individual research articles on psychotherapy for social anxiety disorder in adults.

**Table:**

Database	Medline, PreMEDLINE, PsycINFO (via the OVID SP Interface)
Date	Aug 10, 2018
Searchers	NY/YK

Database search results.

**Table:**

#	Search formula	Number of hits
1	avoidant personality disorder/or hyperhidrosis/or mutism/or social phobia/	28 469
2	blushing/or exp hyperhidrosis/or mutism/or phobic disorders/or shyness/	16 957
3	avoidant personality disorder/or elective mutism/or social anxiety/or social phobia/or sweating/or timidity/	107 278
4	(((anxiet$ or anxious$ or phobia$ or phobic$) adj2 (performance or social$)) or socioanxi$ or sociophobi$ or ((blush$ or sweat$ or trembl$) adj3 (anxiet$ or anxious$ or chronic$ or excessiv$ or fear$ or severe)) or ((interpersonal or inter personal or social$ or socio$) adj2 (aversion$ or aversiv$ or confiden$ or difficult$ or disorder$ or distress$ or fear$)) or hyperhydrosis or hyperperspirat$ or (hyper adj (hydrosis or perspirat$)) or ((mute$ or mutism) adj2 (elective$ or selective$)) or ((negative evaluation or speak$) adj3 (anxiet$ or anxious$ or distress$ or fear$)) or paruresis or (((personalit$ or phobi$ or social$ or socio$) adj2 avoid$) or avoidant disorder) or (phobi$ adj2 neuros$) or phobic disorder$ or (school$ adj2 (anxiet$ or anxious$ or phobi$ or refuse or refusal)) or (shy or shyness) or specific phobia$).ti,ab.	57 439
5	1 or 2 or 3 or 4	158 917
6	limit 5 to “300 adulthood <age 18 years and older> “[Limit not valid in Ovid MEDLINE(R),Ovid MEDLINE(R) Daily Update,Ovid MEDLINE(R) In‐Process; records were retained]	141 200
7	limit 6 to humans [Limit not valid in PsycINFO; records were retained]	128 324
8	limit 7 to (“reviews (maximizes specificity)” or “therapy (maximizes specificity)”)	9954
9	limit 8 to journal article	9807
10	limit 9 to adulthood <18+ years> [Limit not valid in Ovid MEDLINE(R),Ovid MEDLINE(R) Daily Update,Ovid MEDLINE(R) In‐Process,Ovid MEDLINE(R) Publisher; records were retained]	9607
11	limit 10 to (english or japanese)	9516
12	limit 11 to (“0300 clinical trial” or “0451 prospective study” or “0830systematic review” or 1200 meta analysis) [Limit not valid in Ovid MEDLINE(R),Ovid MEDLINE(R) Daily Update,Ovid MEDLINE(R) In‐Process,Ovid MEDLINE(R) Publisher; records were retained]	8945
13	limit 12 to human	8943
14	limit 13 to (clinical trial, all or meta analysis) [Limit not valid in PsycINFO; records were retained]	7869
15	limit 14 to year = “2016–2018”	1261

### CQ3: What are the recommended treatments for social anxiety disorder in adults in terms of psychotherapy and pharmacotherapy, and monotherapy and combination therapy?

Title: Search for SR/MA articles and individual research articles on psychotherapy, pharmacotherapy, and monotherapy and combination therapy, respectively, for social anxiety disorder in adults.

**Table:**

Database	Medline, PreMEDLINE, PsycINFO (via the OVID SP Interface)
Date	Aug 10, 2018
Searchers	NY/YK

Database search results

**Table:**

#	Search formula	Number of hits
1	avoidant personality disorder/or hyperhidrosis/or mutism/or social phobia/	28 469
2	blushing/or exp hyperhidrosis/or mutism/or phobic disorders/or shyness/	16 957
3	avoidant personality disorder/or elective mutism/or social anxiety/or social phobia/or sweating/or timidity/	107 278
4	(((anxiet$ or anxious$ or phobia$ or phobic$) adj2 (performance or social$)) or socioanxi$ or sociophobi$ or ((blush$ or sweat$ or trembl$) adj3 (anxiet$ or anxious$ or chronic$ or excessiv$ or fear$ or severe)) or ((interpersonal or inter personal or social$ or socio$) adj2 (aversion$ or aversiv$ or confiden$ or difficult$ or disorder$ or distress$ or fear$)) or hyperhydrosis or hyperperspirat$ or (hyper adj (hydrosis or perspirat$)) or ((mute$ or mutism) adj2 (elective$ or selective$)) or ((negative evaluation or speak$) adj3 (anxiet$ or anxious$ or distress$ or fear$)) or paruresis or (((personalit$ or phobi$ or social$ or socio$) adj2 avoid$) or avoidant disorder) or (phobi$ adj2 neuros$) or phobic disorder$ or (school$ adj2 (anxiet$ or anxious$ or phobi$ or refuse or refusal)) or (shy or shyness) or specific phobia$).ti,ab.	57 439
5	1 or 2 or 3 or 4	158 917
6	limit 5 to “300 adulthood <age 18 years and older> “[Limit not valid in Ovid MEDLINE(R),Ovid MEDLINE(R) Daily Update,Ovid MEDLINE(R) In‐Process; records were retained]	141 200
7	limit 6 to humans [Limit not valid in PsycINFO; records were retained]	128 324
8	limit 7 to (“reviews (maximizes specificity)” or “therapy (maximizes specificity)”)	9954
9	limit 8 to journal article	9807
10	limit 9 to adulthood <18+ years> [Limit not valid in Ovid MEDLINE(R),Ovid MEDLINE(R) Daily Update,Ovid MEDLINE(R) In‐Process,Ovid MEDLINE(R) Publisher; records were retained]	9607
11	limit 10 to (english or japanese)	9516
12	limit 11 to (“0300 clinical trial” or “0451 prospective study” or “0830systematic review” or 1200 meta analysis) [Limit not valid in Ovid MEDLINE(R),Ovid MEDLINE(R) Daily Update,Ovid MEDLINE(R) In‐Process,Ovid MEDLINE(R) Publisher; records were retained]	8945
13	limit 12 to human	8943
14	limit 13 to (clinical trial, all or meta analysis) [Limit not valid in PsycINFO; records were retained]	7869
15	limit 14 to year = “2016–2018”	1261

### Postnote

A three‐arm, double‐blind, randomized, controlled trial of escitalopram (Lexapro®) in patients with social anxiety disorder in Japan using 10‐mg escitalopram, 20‐mg escitalopram, and placebo groups with change in LSAS‐J total score at 12 weeks as the primary endpoint^1^ is presented here as reference information because details of the efficacy validation study have been reported in the Mochida Pharmaceutical Lexapro® Pharmaceutical Interview Form (April 2020, revised January 2021 [11th edition]; the figures in parentheses below are quoted from the Pharmaceutical Interview Form). The analysis plan is the following: “The primary endpoint (change in LSAS‐J total score [last observation carried forward] at 12 weeks) will be compared between treatment groups using analysis of covariance (ANCOVA), with treatment group as a factor and baseline LSAS‐J total score as a covariate. The 20‐mg escitalopram group will be compared with the placebo group only if the superiority of the 10‐mg escitalopram group to the placebo group is verified. The change in LSAS‐J total score will be analyzed. The change in LSAS‐J total score (at 12 weeks) will be analyzed by observed case and mixed‐effect model repeated measure (MMRM) as a sensitivity analysis, and the change in LSAS‐J total score (observed case) will be analyzed.” As a result, the LSAS‐J total score (primary endpoint) did not show a statistically significant difference between the 10‐mg escitalopram and placebo groups with regard to change in LSAS‐J total score (full analysis set, last observation carried forward) at week 12. Therefore, the 20‐mg escitalopram group hypothesis was not tested. However, a non‐multiple post hoc analysis showed a statistically significant difference in change in LSAS‐J total scores between the placebo and 20‐mg escitalopram groups. The MMRM and observed case analyses were performed for sensitivity analysis of the change in LSAS‐J total scores at 12 weeks (full analysis set). The results of the MMRM analysis showed a statistically significant difference in the mean change in LSAS‐J total score (at 12 weeks) between the placebo and 10‐mg escitalopram groups and between the placebo and 20‐mg escitalopram groups. The ANCOVA results of the observed case analysis showed a statistically significant difference in the mean change between the placebo group and 10‐ and 20‐mg escitalopram groups.

In the meta‐analysis that was conducted for the guideline, each article was included after a careful examination of whether it met the inclusion criteria, after which the extent of risk of bias was examined for each article, and the data from each article were used to analyze the response rate and reduction of social anxiety symptoms (LSAS reduction). As such, the authors' interpretations of the conclusions of the Asakura et al.^1^ paper above were not reflected in the systematic review.
