# Supplementary material for: Japanese Society of Anxiety and Related Disorders/Japanese Society of Neuropsychopharmacology: Clinical practice guideline for social anxiety disorder (2021)
Source: Neuropsychopharmacol Rep. 2023 Aug 25;43(3):288–309. doi: 10.1002/npr2.12365 (PMC10496046; doi:10.1002/npr2.12365)
Supplement: Supplementary file 1 — Appendix S1 [file NPR2-43-288-s001.docx]

**Supplementary Information**

**CQ1: What is the recommended pharmacotherapy for social anxiety disorder in adults?**

**1. Pharmacotherapy other than SSRIs and SNRIs**

SSRIs and venlafaxine^*^, which is classified as an SNRI, have been proposed as pharmacological treatments for social anxiety disorder in adults, but there are currently three drugs for social anxiety disorder that are covered by insurance in Japan: fluvoxamine, paroxetine, and escitalopram. Treatment response rates for single-agent pharmacotherapy with each of these drugs are 43-48%, 55-72%, and 54-71%, respectively, and remission rates are not very high, ranging from 10% to 30%.^1^ Even if we assume a 70% response rate for each drug and a 30% remission rate, which is the consensus among existing guidelines (NICE in the UK,^2^ S3 in Germany,^3^ and Canadian CPG in Canada^4^), then we can infer that at least 10% (0.3 × 0.3) of patients do not respond to treatment, and the remission rate is estimated to be only 50% at best (0.3 + 0.7 × 0.3).

Since the previous systematic review,^5^ there have been no new RCTs of any classes of drugs other than SSRIs or SNRIs (antiepileptics and analogs, antipsychotics, benzodiazepines, beta blockers, MAOIs, NARIs, NaSSAs, RIMAs, SARIs, and other antidepressants). Because none of these drugs have been adequately studied, they have not been evaluated for recommendations in this guideline. However, they may be an option for patients who are difficult to treat with SSRIs or SNRIs and are reviewed here with reference to previous guidelines and systematic reviews (Drugs not yet marketed in Japan or not approved for social anxiety disorder are indicated with an asterisk [^*^] next to their name).

**1.1. Antiepileptics and analogs**

Pregabalin^*^ is the non-standard treatment in S3 and first-line treatment in Canadian CPG. Gabapentin^*^ is the non-standard treatment in S3 and second-line treatment in Canadian CPG. Levetiracetam^*^ is the non-standard treatment in S3 and not recommended in Canadian CPG. None of them are recommended in the NICE guideline. In Japan, pregabalin^*^ is indicated for neuropathic pain and fibromyalgia, gabapentin^*^ is indicated for the adjunctive treatment of epilepsy, and levetiracetam^*^ is indicated for the adjunctive treatment of partial-onset seizures and tonic-clonic seizures.

In two RCTs of pregabalin^*^ (*N* = 463),^6, 7^ both studies showed efficacy against placebo at 600 mg/day but no efficacy against placebo at lower doses. A study by Feltner et al.^6^ reported a high dropout rate (14-20.5% *vs*. 1.2% for placebo) because of side effects of pregabalin^*^. One small (*N* = 69) RCT of gabapentin^*8^ demonstrated efficacy. A meta-analysis of three studies of pregabalin^*^ and gabapentin^*^ also showed efficacy (relative risk [RR] = 1.60; 95% confidence interval [CI] = 1.16 to 2.20, *N* = 532).

Two RCTs of levetiracetam^*9, 10^ showed no benefit with regard to efficacy against placebo (RR = 0.98; 95% CI = 0.70 to 1.37, *N* = 228).

**1.2. Antipsychotics**

Olanzapine^*^ is listed as a non-standard treatment in S3 and a third-line treatment in Canadian CPG, but it is not recommended in the NICE guideline. In Japan, olanzapine^*^ is indicated for the treatment of schizophrenia and bipolar disorder.

Only one very small RCT (*N* = 12) of olanzapine^*11^ has been conducted, and its efficacy has been reported, but there is a lack of evidence.

**1.3. Benzodiazepines**

Benzodiazepines are not recommended in the NICE guideline. They are only recommended in S3 when standard therapies prove ineffective, and they are second-line treatments in Canadian CPG.

For benzodiazepines, a meta-analysis of RCTs of clonazepam^*12^ and bromazepam^13^ showed high efficacy (RR = 4.03; 95% CI = 2.45 to 6.65, *N* = 132). However, both were small RCTs, and there have been no RCTs of benzodiazepines in recent years. Based on their empirical clinical usefulness, benzodiazepines are currently often prescribed for social anxiety disorder.^14^ The dropout rate because of short-term side effects is low,^15^ but in the long term, there are problems of dependence and tolerance,^16^ and the use of benzodiazepines should be carefully considered in light of the risks.

**1.4. Beta blockers**

NICE, S3, and Canadian CPG do not recommend beta blockers, and there has been insufficient evidence of their efficacy to date.

**1.5. Monoamine oxidase inhibitors (MAOIs)**

MAOIs are third-line drugs in the NICE guideline and not recommended in S3. Phenelzine^*^ is a second-line drug in Canadian CPG. Selegiline^*^ is a third-line drug in Canadian CPG. This is attributable to drug interactions, the need for dietary restriction, liver damage, hypertension, and other side effects. The only drug that is classified as an MAOI and marketed in Japan is selegiline^*^ (indicated for Parkinson’s disease), which is an MAO-B inhibitor.

A meta-analysis of four RCTs of phenelzine^*17-20^ showed efficacy (RR = 2.36; 95% CI = 1.48 to 3.75, *N* = 235). Regardless of its efficacy, however, side effects and drug interactions of phenelzine have led to its not being highly recommended in prior guidelines.

For selegiline^*^, only a small (*N* = 16) open-label study^21^ showed efficacy.

**1.6. Norepinephrine reuptake inhibitors (NARIs)**

NARIs are not recommended in the NICE guideline or S3, and atomoxetine^*^ is a third-line drug in the Canadian CPG. In Japan, atomoxetine^*^ is marketed for the treatment of attention-deficit/hyperactivity disorder (ADHD).

There is only one RCT (*N* = 27) on the NARI atomoxetine^*^,^22^ which showed no efficacy compared with placebo (RR = 0.70; 95% CI = 0.19 to 2.54).

**1.7. Noradrenergic and specific serotonergic antidepressants (NaSSAs)**

NaSSAs are not recommended in the NICE guideline, are non-standard treatments in S3, and are third-line treatments in the Canadian CPG. Mirtazapine^*^ is marketed in Japan for the treatment of depression.

There are two small RCTs of mirtazapine^*^, which is classified as a NaSSA and has not yielded consistent results. One study showed efficacy,^23^ whereas the other^24^ showed no benefit.

**1.8. Reversible inhibitors of monoamine oxidase A (RIMAs)**

RIMAs are third-line treatments in the NICE guideline. The expert consensus in S3 is that RIMAs are effective for social anxiety disorder, and they are third-line treatments in the Canadian CPG. RIMAs have not yet been marketed in Japan.

Five non-small RCTs of moclobemide^*^, a RIMA, showed efficacy (RR = 1.32; 95% CI = 1.14 to 1.52, *N* = 1063).^25-29^ Long-term efficacy was also reported in one study (RR = 1.50; 95% CI = 1.12 to 2.00, *N* = 90).^29^

**1.9. Serotonin 2 antagonist and reuptake inhibitors (SARIs)**

The preceding guidelines do not make any recommendations about SARIs. Although the SARI trazodone^*^ is marketed in Japan as an antidepressant, there have been no RCTs to date for social anxiety disorder, and its efficacy has not been verified.

**1.10. Other antidepressants**

A small RCT for social anxiety disorder and comorbid depression was conducted using another antidepressant, vortioxetine^*^.^30^ It has shown efficacy for both depression and social anxiety disorder symptoms compared with placebo.

**1.11. Combination therapy**

A combination of paroxetine and clonazepam^*^ was reported to be more effective than each monotherapy alone, although the sample size was small (*N* = 14).^31^ An RCT (*N* = 397) of social anxiety disorder patients who were unresponsive to the SSRI sertraline^*^ found that the combination of the benzodiazepine clonazepam^*^ with the SSRI sertraline^*^ was more effective than either monotherapy alone, although the sample size was small (*N* = 14).^32^ In the same study, the substitution of an SSRI with an SNRI (venlafaxine^*^) and the addition of a placebo to an SSRI resulted in significantly higher response rates with the benzodiazepine addition than with placebo for SSRIs. Although neither study considered difficulties of the long-term dose reduction of benzodiazepines, the addition of benzodiazepines may be an option for patients who do not respond to SSRIs.

Drugs other than SSRIs and SNRIs that have been shown to be effective for social anxiety disorder in multiple RCTs with very weak evidence include benzodiazepines, phenelzine^*^, moclobemide^*^, and pregabalin^*^, although only benzodiazepines and pregabalin^*^ are marketed in Japan. Notably, the only indication for clonazepam^*^, a benzodiazepine, is epilepsy. Other benzodiazepines are indicated for anxiety, tension, and depression in neurosis, but there is little evidence of their efficacy for social anxiety disorder, and they should be used with caution by considering tolerance and their dependence potential. Pregabalin^*^ is indicated for neuropathic pain and fibromyalgia but not for social anxiety disorder.

The strength of evidence for drugs other than SSRIs and SNRIs is very weak, and any consideration of their use should be done with caution by considering their indications, risks, and benefits.

**2. Pharmacotherapy for social anxiety disorder with comorbidities**

The lifetime prevalence of social anxiety disorder has been reported to be 2-12% in large epidemiological studies,^33^ and high rates of comorbidity with other psychiatric disorders have been reported. An epidemiologic study of 4174 adults in Germany reported that of 83 individuals who met the criteria for social anxiety disorder, 87.8% had other comorbid psychiatric disorders, with comorbidity rates of 71.2% for other anxiety disorders, 50.5% for depression, 38.1% for dysthymia, and 5.7% for bipolar disorder. All of these prevalence rates have 10-times higher odds ratios than patients without social anxiety disorder.^34^ The high prevalence of comorbidities is consistent with a higher prevalence of social anxiety disorder, and it may make drug selection for social anxiety disorder more difficult. Pharmacotherapy for social anxiety disorder with comorbidities are discussed below (Drugs not yet marketed in Japan or not approved for social anxiety disorder are indicated with an asterisk [^*^] next to their name).

**2.1. Pharmacotherapy for comorbid bipolar disorder**

In an epidemiological study of 8091 adults in the United States, the odds ratios of patients with social anxiety disorder with comorbidities were 2.9 for depression, 2.7 for dysthymia, and 5.9 for bipolar disorder, suggesting that a high prevalence of social anxiety disorder symptoms is a risk factor for comorbid mood disorders.^35^

Although comorbid depression is more resistant to treatment, the standard treatment of social anxiety disorder does not conflict significantly with the treatment of depression, and pharmacotherapy can be considered in accordance with drug selection for both disorders.^36^ With comorbid bipolar disorder, drug selection becomes difficult because SSRIs and SNRIs, which are first-line treatments for social anxiety disorder, are not generally recommended for bipolar disorder, and antidepressants may destabilize phases of bipolar disorder.^37^ The leading guidelines from the APA in the United States,^38^ Canadian Network for Mood Disorders (CANMAT) in Canada,^39^ NICE in the United Kingdom,^40^ and World Federation of Societies of Biological Psychiatry^41^ do not recommend monotherapy with antidepressants, even if the patient is in the depressive phase. The latest CANMAT guideline recommends a stepwise approach to the treatment of bipolar disorder with comorbid anxiety, with priority given to mood stabilization first, followed by the treatment of anxiety symptoms. Pregabalin^*^ and lorazepam, which do not destabilize mood, are recommended for pharmacotherapy, and if lorazepam is used, then it should only be used for short periods of time to avoid dependence. Because antidepressants, especially SNRIs, increase the risk of mania, SSRIs are the first choice when antidepressants are used. Even with SSRIs, adequate phase prophylaxis with one or more mood stabilizers (lithium, valproate, second-generation antipsychotics, etc.) is recommended.

There have been no RCTs of pharmacotherapy for solely social anxiety disorder with comorbid bipolar disorder, but there have been several RCTs for other comorbid anxiety and obsessive-compulsive disorders and posttraumatic stress disorder (PTSD).

An RCT was conducted with olanzapine and lamotrigine in 47 patients with bipolar I or II disorder who had been in remission with lithium for at least 2 months and had various comorbid anxiety symptoms (panic, social anxiety disorder, specific phobia, obsessive-compulsive disorder, and generalized anxiety).^42^ Both medications significantly reduced anxiety symptoms compared with baseline, with significantly greater reductions with olanzapine than with lamotrigine.

There are also several RCTs for sub-diagnostic threshold anxiety symptoms that are comorbid with bipolar disorder.

A small (*N* = 25) RCT of the antidepressant and anxiolytic effects of valproate on major depressive episodes of bipolar I disorder was conducted.^43^ Both depressive and anxiety symptoms significantly decreased compared with placebo, suggesting valproate’s efficacy.

An RCT of olanzapine monotherapy, a olanzapine/fluoxetine^*^ fixed-dose combination, and placebo was conducted in patients with bipolar I disorder (*N* = 833) during a major depressive episode. A *post hoc* analysis of the results was conducted to assess the efficacy of these treatments for comorbid anxiety symptoms.^44^ Patients were divided into two groups: one with comorbid anxiety and one without comorbid anxiety. The comorbid group had more severe depressive symptoms at baseline and lower response and remission rates at the end of 8 weeks compared with the non-comorbid group, but there was no difference in the amount of change in depressive symptoms. Both olanzapine monotherapy and the olanzapine/fluoxetine^*^ fixed-dose combination showed significant improvement in depressive and anxiety symptoms compared with placebo, with a trend toward greater improvement with the olanzapine/fluoxetine^*^ fixed-dose combination, regardless of the presence of comorbid anxiety.

Data from two large clinical trials (*N* = 978) that examined the efficacy of quetiapine^*^ for major depressive episodes in bipolar I and II disorders were pooled, and *post hoc* analyses were performed for anxiety symptoms.^45^ The results showed a significant reduction of anxiety symptoms compared with placebo, indicating efficacy in reducing anxiety symptoms. Additionally, an analysis of baseline anxiety intensity in the high-anxiety, moderate-anxiety, and low-anxiety groups showed that baseline anxiety intensity was not associated with improvement in depressive symptoms and did not affect the antidepressant effect of quetiapine^*^.

As described above, there is a lack of evidence of the pharmacotherapy of bipolar disorder with social anxiety disorder comorbidity, and empiric pharmacotherapy should be used with reference to findings in the treatment of anxiety symptoms in patients with bipolar disorder. In the case of social anxiety disorder that is comorbid with or suspected of being comorbid with bipolar disorder, the patient should be treated with mood stabilizers or second-generation antipsychotics to first stabilize depressive and (hypo)manic phases in accordance with the pharmacotherapy for bipolar disorder. The mood stabilizers valproate and lamotrigine and second-generation antipsychotics olanzapine and quetiapine^*^ may reduce anxiety.

If social anxiety symptoms are problematic despite a stable bipolar phase, then the use of SSRIs may be an option and should be considered in combination with mood stabilizers or second-generation antipsychotics.

As mentioned earlier, benzodiazepines have also been shown to be effective in the treatment of social anxiety disorder, but the lifetime prevalence of substance dependence in bipolar disorder is as high as 40% or more,^46^ and comorbid drug dependence has been reported to worsen prognosis.^47^ There have also been reports of no symptom improvement in bipolar patients who are treated with lithium and quetiapine^*^ when benzodiazepines are added to their treatment regimen.^48^ Therefore, the use of benzodiazepines should be limited to cases where short-term use can offer sufficient improvement while considering dependence and tolerance.

Pregabalin^*^, which has been shown to be effective in multiple RCTs for social anxiety disorder, is also a candidate because it does not appear to destabilize the bipolar phase of the disorder, but its efficacy in patients with comorbid bipolar disorder has not been reported, and it is not covered by insurance in Japan.

**2.2. Pharmacotherapy for comorbid schizophrenia**

Social anxiety disorder with schizophrenia comorbidity has been reported to increase the risk of suicide and alcohol abuse and reduce social adjustment and quality of life.^49^ Although no RCTs have been conducted for social anxiety disorder that is comorbid with schizophrenia, small open-label trials for social anxiety symptoms have been reported.

Social anxiety symptoms, quality of life, and psychotic symptoms were compared before and after the replacement of antipsychotic medication with aripiprazole in 16 patients with schizophrenia or schizoaffective disorder who presented with social anxiety symptoms.^50^ The results showed significant improvement in social anxiety symptoms, quality of life, and psychotic symptoms at 2 months before and after replacement, and 10 patients maintained improvement at 1 year after continued follow-up.

Twelve patients with schizophrenia who developed social anxiety symptoms during clozapine treatment were treated with fluoxetine^*^, an SSRI, and 8 of 12 patients reported improvements in social anxiety symptoms at 12 weeks.^51^

Others have reported a reduction of nonspecific anxiety symptoms of schizophrenia with risperidone,^52^ quetiapine,^53^ and olanzapine.^54^ However, all of these studies were open-label or relatively small RCTs and focused on anxiety symptoms below the diagnostic threshold. There is currently no sufficient consensus on SSRI adjunctive therapy with antipsychotics.^55^

**2.3. Pharmacotherapy for comorbid autism spectrum disorder**

In a study of 112 patients with autism spectrum disorder (ASD),^56^ 41.9% had comorbid anxiety, and social anxiety disorder was the most frequent comorbidity (29.2%). Patients with ASD have innate problems with communication and interpersonal relationships, and social anxiety disorder is thought to be the most common comorbid anxiety disorder.

Currently, no medications are effective for communication disorders in ASD. SSRIs may reduce stereotypic behaviors, but the research results are inconsistent.^57^ Diagnostic criteria for social anxiety disorder in the DSM-5 exclude social anxiety symptoms that can be explained as ASD symptoms from the social anxiety disorder diagnosis.^58^ Social anxiety symptoms as secondary symptoms of ASD and social anxiety symptoms of social anxiety disorder without ASD are considered to have different etiologies. Therefore, there is little evidence to support the application of evidence that is obtained in social anxiety disorder to the treatment of social anxiety symptoms as secondary symptoms of ASD. There is also currently little evidence to suggest that the standard treatment of social anxiety disorder should be attempted for social anxiety symptoms as secondary symptoms of ASD. Therefore, pharmacotherapy should be considered more cautiously for these patients.

**2.4. Pharmacotherapy for comorbid ADHD**

It has been reported that 29.3% of adults with ADHD have comorbid social anxiety disorder, making it the most frequent anxiety disorder that is comorbid with ADHD.^59^ ADHD medications include the selective NARI atomoxetine and neurostimulant methylphenidate. Atomoxetine is the subject of an RCT for social anxiety disorder.

An RCT of atomoxetine and placebo in adult patients with social anxiety disorder with comorbid ADHD (*N* = 442) was conducted.^60^ Atomoxetine significantly improved both ADHD and social anxiety disorder symptoms compared with placebo. Another RCT of atomoxetine in adult social anxiety disorder patients without comorbid ADHD did not show efficacy with regard to social anxiety disorder symptoms.^22^ Although the reports are inconsistent, atomoxetine may be an option for social anxiety disorder with comorbid ADHD.

Only one case series of methylphenidate for adult ADHD patients with comorbid social anxiety disorder has been reported. A case series of 20 patients on extended-release methylphenidate showed that only three patients dropped out of treatment, and 17 patients showed improvement in ADHD and social anxiety disorder symptoms.^61^ Further RCTs are needed to validate the efficacy of methylphenidate.

No RCTs of combination therapy with atomoxetine or methylphenidate and other drugs have been reported. Venlafaxine^*^, an SNRI, was not significantly different from placebo in improving ADHD symptoms in an RCT of adults with ADHD,^62^ but it did show a significant difference in response rate, suggesting that venlafaxine may be an option for patients with comorbid social anxiety disorder.

In summary, little is known about pharmacotherapy for social anxiety disorder with other comorbid psychiatric disorders, and pharmacotherapy has not yet been established. We must make drug selections based on experience by considering standard treatments of social anxiety disorder and comorbid psychiatric disorders.

References for Supplementary Information CQ1:

1. Stein MB, Stein DJ. Social anxiety disorder. Lancet. 2008;371(9618):1115-25.

2. National Institute for Health and Care Excellence (NICE). Social anxiety disorder: recognition, assessment and treatment (Clinical guideline [CG159]): Leicester (UK): British Psychological Society; 2013 Available from: http://guidance.nice.org.uk/CG159.

3. Bandelow B, Lichte T, Rudolf S, Wiltink J, Beutel ME. The diagnosis of and treatment recommendations for anxiety disorders. Dtsch Arztebl Int. 2014;111(27-28):473-80.

4. Katzman MA, Bleau P, Blier P, Chokka P, Kjernisted K, Van Ameringen M, et al. Canadian clinical practice guidelines for the management of anxiety, posttraumatic stress and obsessive-compulsive disorders. BMC Psychiatry. 2014;14 Suppl 1(Suppl 1):S1.

5. Williams T, Hattingh CJ, Kariuki CM, Tromp SA, van Balkom AJ, Ipser JC, et al. Pharmacotherapy for social anxiety disorder (SAnD). Cochrane Database Syst Rev. 2017;10(10):Cd001206.

6. Feltner DE, Liu-Dumaw M, Schweizer E, Bielski R. Efficacy of pregabalin in generalized social anxiety disorder: results of a double-blind, placebo-controlled, fixed-dose study. Int Clin Psychopharmacol. 2011;26(4):213-20.

7. Pande AC, Feltner DE, Jefferson JW, Davidson JR, Pollack M, Stein MB, et al. Efficacy of the novel anxiolytic pregabalin in social anxiety disorder: a placebo-controlled, multicenter study. J Clin Psychopharmacol. 2004;24(2):141-9.

8. Pande AC, Davidson JR, Jefferson JW, Janney CA, Katzelnick DJ, Weisler RH, et al. Treatment of social phobia with gabapentin: a placebo-controlled study. J Clin Psychopharmacol. 1999;19(4):341-8.

9. Stein MB, Ravindran LN, Simon NM, Liebowitz MR, Khan A, Brawman-Mintzer O, et al. Levetiracetam in generalized social anxiety disorder: a double-blind, randomized controlled trial. J Clin Psychiatry. 2010;71(5):627-31.

10. Zhang W, Connor KM, Davidson JR. Levetiracetam in social phobia: a placebo controlled pilot study. J Psychopharmacol. 2005;19(5):551-3.

11. Barnett SD, Kramer ML, Casat CD, Connor KM, Davidson JR. Efficacy of olanzapine in social anxiety disorder: a pilot study. J Psychopharmacol. 2002;16(4):365-8.

12. Davidson JR, Potts N, Richichi E, Krishnan R, Ford SM, Smith R, et al. Treatment of social phobia with clonazepam and placebo. J Clin Psychopharmacol. 1993;13(6):423-8.

13. Versiani M, Nardi AE, Figueira I, Mendlowicz M, Marques C. Double-blind placebo controlled trial with bromazepam in social phobia. J Bras Psiquiatr. 1997;46(3):167-71.

14. Roy-Byrne P. Treatment in nonresponsive patients with social anxiety: back to the future with benzodiazepines. Am J Psychiatry. 2014;171(1):1-4.

15. Connor KM, Davidson JR, Potts NL, Tupler LA, Miner CM, Malik ML, et al. Discontinuation of clonazepam in the treatment of social phobia. J Clin Psychopharmacol. 1998;18(5):373-8.

16. Schneier FR. Pharmacotherapy of social anxiety disorder. Expert Opin Pharmacother. 2011;12(4):615-25.

17. Blanco C, Heimberg RG, Schneier FR, Fresco DM, Chen H, Turk CL, et al. A placebo-controlled trial of phenelzine, cognitive behavioral group therapy, and their combination for social anxiety disorder. Arch Gen Psychiatry. 2010;67(3):286-95.

18. Heimberg RG, Liebowitz MR, Hope DA, Schneier FR, Holt CS, Welkowitz LA, et al. Cognitive behavioral group therapy vs phenelzine therapy for social phobia: 12-week outcome. Arch Gen Psychiatry. 1998;55(12):1133-41.

19. Liebowitz MR, Schneier F, Campeas R, Hollander E, Hatterer J, Fyer A, et al. Phenelzine vs atenolol in social phobia. A placebo-controlled comparison. Arch Gen Psychiatry. 1992;49(4):290-300.

20. Versiani M, Nardi AE, Mundim FD, Alves AB, Liebowitz MR, Amrein R. Pharmacotherapy of social phobia. A controlled study with moclobemide and phenelzine. Br J Psychiatry. 1992;161:353-60.

21. Simpson HB, Schneier FR, Marshall RD, Campeas RB, Vermes D, Silvestre J, et al. Low dose selegiline (L-Deprenyl) in social phobia. Depress Anxiety. 1998;7(3):126-9.

22. Ravindran LN, Kim DS, Letamendi AM, Stein MB. A randomized controlled trial of atomoxetine in generalized social anxiety disorder. J Clin Psychopharmacol. 2009;29(6):561-4.

23. Muehlbacher M, Nickel MK, Nickel C, Kettler C, Lahmann C, Pedrosa Gil F, et al. Mirtazapine treatment of social phobia in women: a randomized, double-blind, placebo-controlled study. J Clin Psychopharmacol. 2005;25(6):580-3.

24. Schutters SI, Van Megen HJ, Van Veen JF, Denys DA, Westenberg HG. Mirtazapine in generalized social anxiety disorder: a randomized, double-blind, placebo-controlled study. Int Clin Psychopharmacol. 2010;25(5):302-4.

25. Katschnig H, Stein M, Buller R, on behalf of the International Multicenter Clinical Trial Group on Moclobeminde in Social Phobia. The International Multicenter Clinical Trial Group on Moclobemide in Social Phobia. Moclobemide in social phobia. A double-blind, placebo-controlled clinical study. Eur Arch Psychiatry Clin Neurosci. 1997;247(2):71-80.

26. Noyes R, Jr., Moroz G, Davidson JR, Liebowitz MR, Davidson A, Siegel J, et al. Moclobemide in social phobia: a controlled dose-response trial. J Clin Psychopharmacol. 1997;17(4):247-54.

27. Oosterbaan DB, Balkom AJ, Spinhoven P, Oppen PV, Dyck RV. Cognitive therapy versus moclobemide in social phobia: a controlled study. Clin Psychol Psychother. 2001;8(4):263-73.

28. Schneier FR, Goetz D, Campeas R, Fallon B, Marshall R, Liebowitz MR. Placebo-controlled trial of moclobemide in social phobia. Br J Psychiatry. 1998;172:70-7.

29. Stein DJ, Cameron A, Amrein R, Montgomery SA. Moclobemide is effective and well tolerated in the long-term pharmacotherapy of social anxiety disorder with or without comorbid anxiety disorder. Int Clin Psychopharmacol. 2002;17(4):161-70.

30. Liebowitz MR, Careri J, Blatt K, Draine A, Morita J, Moran M, et al. Vortioxetine versus placebo in major depressive disorder comorbid with social anxiety disorder. Depress Anxiety. 2017;34(12):1164-72.

31. Seedat S, Stein MB. Double-blind, placebo-controlled assessment of combined clonazepam with paroxetine compared with paroxetine monotherapy for generalized social anxiety disorder. J Clin Psychiatry. 2004;65(2):244-8.

32. Pollack MH, Van Ameringen M, Simon NM, Worthington JW, Hoge EA, Keshaviah A, et al. A double-blind randomized controlled trial of augmentation and switch strategies for refractory social anxiety disorder. Am J Psychiatry. 2014;171(1):44-53.

33. Tsuchiya M, Kawakami S. Epidemiology of social anxiety disorders. Jpn J Clin Psychiatry. 2007;36(12):1495−502. [in Japanese]

34. Fehm L, Beesdo K, Jacobi F, Fiedler A. Social anxiety disorder above and below the diagnostic threshold: prevalence, comorbidity and impairment in the general population. Soc Psychiatry Psychiatr Epidemiol. 2008;43(4):257-65.

35. Kessler RC, Stang P, Wittchen HU, Stein M, Walters EE. Lifetime co-morbidities between social phobia and mood disorders in the US National Comorbidity Survey. Psychol Med. 1999;29(3):555-67.

36. Panzer MJ. Are SSRIs really more effective for anxious depression? Ann Clin Psychiatry. 2005;17(1):23-9.

37. McElroy SL, Kotwal R, Kaneria R, Keck PE, Jr. Antidepressants and suicidal behavior in bipolar disorder. Bipolar Disord. 2006;8(5 Pt 2):596-617.

38. American Psychiatric Association. Practice guideline for the treatment of patients with bipolar disorder (revision). Am J Psychiatry. 2002;159(4 Suppl):1-50.

39. Yatham LN, Kennedy SH, Parikh SV, Schaffer A, Bond DJ, Frey BN, et al. Canadian Network for Mood and Anxiety Treatments (CANMAT) and International Society for Bipolar Disorders (ISBD) 2018 guidelines for the management of patients with bipolar disorder. Bipolar Disord. 2018;20(2):97-170.

40. National Institute for Health and Care Excellence (NICE). Bipolar disorder: The management of bipolar disorder in adults, children and adolescents, in primary and secondary care (Clinical guideline [CG38]): Leicester (UK): British Psychological Society; 2006 Available from: https://www.nice.org.uk/guidance/cg38.

41. Grunze H, Vieta E, Goodwin GM, Bowden C, Licht RW, Möller HJ, et al. The World Federation of Societies of Biological Psychiatry (WFSBP) guidelines for the biological treatment of bipolar disorders: update 2012 on the long-term treatment of bipolar disorder. World J Biol Psychiatry. 2013;14(3):154-219.

42. Maina G, Albert U, Rosso G, Bogetto F. Olanzapine or lamotrigine addition to lithium in remitted bipolar disorder patients with anxiety disorder comorbidity: a randomized, single-blind, pilot study. J Clin Psychiatry. 2008;69(4):609-16.

43. Davis LL, Bartolucci A, Petty F. Divalproex in the treatment of bipolar depression: a placebo-controlled study. J Affect Disord. 2005;85(3):259-66.

44. Tohen M, Calabrese J, Vieta E, Bowden C, Gonzalez-Pinto A, Lin D, et al. Effect of comorbid anxiety on treatment response in bipolar depression. J Affect Disord. 2007;104(1-3):137-46.

45. Lydiard RB, Culpepper L, Schiöler H, Gustafsson U, Paulsson B. Quetiapine monotherapy as treatment for anxiety symptoms in patients with bipolar depression: a pooled analysis of results from 2 double-blind, randomized, placebo-controlled studies. Prim Care Companion J Clin Psychiatry. 2009;11(5):215-25.

46. Cassidy F, Ahearn EP, Carroll BJ. Substance abuse in bipolar disorder. Bipolar Disord. 2001;3(4):181-8.

47. Salloum IM, Thase ME. Impact of substance abuse on the course and treatment of bipolar disorder. Bipolar Disord. 2000;2(3 Pt 2):269-80.

48. Bobo WV, Reilly-Harrington NA, Ketter TA, Brody BD, Kinrys G, Kemp DE, et al. Effect of adjunctive benzodiazepines on clinical outcomes in lithium- or quetiapine-treated outpatients with bipolar I or II disorder: results from the Bipolar CHOICE trial. J Affect Disord. 2014;161:30-5.

49. Pallanti S, Quercioli L, Hollander E. Social anxiety in outpatients with schizophrenia: a relevant cause of disability. Am J Psychiatry. 2004;161(1):53-8.

50. Stern RG, Petti TA, Bopp K, Tobia A. Aripiprazole for the treatment of schizophrenia with co-occurring social anxiety: an open-label cross-taper study. J Clin Psychopharmacol. 2009;29(3):206-9.

51. Pallanti S, Quercioli L, Rossi A, Pazzagli A. The emergence of social phobia during clozapine treatment and its response to fluoxetine augmentation. J Clin Psychiatry. 1999;60(12):819-23.

52. Blin O, Azorin JM, Bouhours P. Antipsychotic and anxiolytic properties of risperidone, haloperidol, and methotrimeprazine in schizophrenic patients. J Clin Psychopharmacol. 1996;16(1):38-44.

53. Kasper S. Quetiapine is effective against anxiety and depressive symptoms in long-term treatment of patients with schizophrenia. Depress Anxiety. 2004;20(1):44-7.

54. Tollefson GD, Sanger TM. Anxious-depressive symptoms in schizophrenia: a new treatment target for pharmacotherapy? Schizophr Res. 1999;35 Suppl:S13-21.

55. Temmingh H, Stein DJ. Anxiety in Patients with Schizophrenia: Epidemiology and Management. CNS Drugs. 2015;29(10):819-32.

56. Simonoff E, Pickles A, Charman T, Chandler S, Loucas T, Baird G. Psychiatric disorders in children with autism spectrum disorders: prevalence, comorbidity, and associated factors in a population-derived sample. J Am Acad Child Adolesc Psychiatry. 2008;47(8):921-9.

57. Lai MC, Lombardo MV, Baron-Cohen S. Autism. Lancet. 2014;383(9920):896-910.

58. American Psychiatric Association. Diagnostic and statistical manual of mental disorders: DSM-5. Washington, DC: American Psychiatric Association; 2013.

59. Kessler RC, Adler L, Barkley R, Biederman J, Conners CK, Demler O, et al. The prevalence and correlates of adult ADHD in the United States: results from the National Comorbidity Survey Replication. Am J Psychiatry. 2006;163(4):716-23.

60. Adler LA, Liebowitz M, Kronenberger W, Qiao M, Rubin R, Hollandbeck M, et al. Atomoxetine treatment in adults with attention-deficit/hyperactivity disorder and comorbid social anxiety disorder. Depress Anxiety. 2009;26(3):212-21.

61. Koyuncu A, Çelebi F, Ertekin E, Kök BE, Tükel R. Extended-release methylphenidate monotherapy in patients with comorbid social anxiety disorder and adult attention-deficit/hyperactivity disorder: retrospective case series. Ther Adv Psychopharmacol. 2017;7(11):241-7.

62. Amiri S, Farhang S, Ghoreishizadeh MA, Malek A, Mohammadzadeh S. Double-blind controlled trial of venlafaxine for treatment of adults with attention deficit/hyperactivity disorder. Hum Psychopharmacol. 2012;27(1):76-81.

**CQ2: What is the recommended psychotherapy (psychological intervention) for social anxiety disorder in adults?**

**Psychotherapy (psychological intervention) not mentioned in the recommendations**

A network meta-analysis of the NICE guideline^1^ showed that the following psychotherapies contributed to improvements in social anxiety symptoms: CBT (individual and group therapy), self-help (with and without support), exposure therapy/social skills training, and short-term psychodynamic therapy. Of these, individual CBT was the most effective and only one of the various treatments, including pharmacotherapy, that outperformed both the waitlist and placebo treatments. Considering these network meta-analysis results and the balance of benefits and harms, the guideline gave the highest recommendation for individual CBT.

This Supplementary Information addresses reasons for the positioning of psychotherapy recommendations other than individual CBT and describes the results of the NICE guideline’s network meta-analysis and Surveillance Report 2017^2^ and other relevant studies.

***Group CBT***

The results of the network meta-analysis and health economic analysis that was conducted in the NICE guideline showed that group CBT is one of the effective treatments for improving social anxiety symptoms, but it is less effective in terms of clinical and health economic effectiveness than individual CBT. Therefore, the recommendation clearly states that individual CBT should be given priority.

***Short-term psychodynamic therapy***

Short-term psychodynamic therapy was shown to be effective in improving social anxiety symptoms in the network meta-analysis that was conducted in the NICE guideline. The NICE guideline also recommends that short-term psychodynamic therapy should be offered to patients who do not wish to receive individual CBT or self-help with support. However, the panel decided not to include this recommendation because there are no standardized manuals or therapist training for short-term psychodynamic therapy specifically for social anxiety disorder in Japan, and it is not part of the insurance reimbursement program.

***Stand-alone techniques, such as exposure therapy and social skills training***

Although these stand-alone techniques were shown to be effective in improving social anxiety symptoms in the network meta-analysis in the NICE guideline, they have often been incorporated into CBT in recent years. Therefore, the panel decided not to recommend these techniques alone, given that they are often included in the most highly recommended techniques of individual CBT.

***Interpersonal therapy and mindfulness-based interventions (mindfulness stress reduction and mindfulness cognitive therapy)***

These psychotherapies were not found to be effective in improving social anxiety symptoms in the network meta-analysis in the NICE guideline compared with the waitlist group.

***Morita therapy***

The network meta-analysis in the NICE guideline did not include any studies on Morita therapy. However, in a subsequent Surveillance Report 2017, Wu et al. (2015)^3^ presented a systematic review of Morita therapy for anxiety-related disorders. This systematic review included seven RCTs that were conducted in China, two of which were conducted for social anxiety disorder (Morita therapy *vs*. pharmacotherapy). However, because of the included trials’ small sizes, inaccuracy, and high risk of bias, no conclusions could be drawn about the effectiveness of Morita therapy in the treatment of anxiety.

References for Supplementary Information CQ2:

1. National Institute for Health and Care Excellence (NICE). Social anxiety disorder: recognition, assessment and treatment (Clinical guideline [CG159]): Leicester (UK): British Psychological Society; 2013 Available from: http://guidance.nice.org.uk/CG159.

2. National Institute for Health and Care Excellence (NICE). Surveillance report 2017 – Social anxiety disorder: recognition, assessment and treatment (2013) NICE guideline CG159: Leicester (UK): British Psychological Society; 2017 Available from: https://www.nice.org.uk/guidance/cg159/resources/surveillance-report-2017-social-anxiety-disorder-recognition-assessment-and-treatment-2013-nice-guideline-cg159-4484818333/chapter/Surveillance-decision?tab=evidence.

3. Wu H, Yu D, He Y, Wang J, Xiao Z, Li C. Morita therapy for anxiety disorders in adults. Cochrane Database Syst Rev. 2015(2):Cd008619.

**CQ3: What are the recommendations regarding monotherapy and combination therapy for social anxiety disorder in adults in terms of pharmacotherapy and psychotherapy (psychological interventions)?**

**1. Rationale for recommendations for combination pharmacotherapy and psychotherapy**

**1-1. Summary and overview of studies of combination therapies that are included in the NICE clinical guideline^1^ and Surveillance Report 2017.^2^**

Among the nine combination therapy studies, psychotherapy was included as group CBT in three studies, supported computerized CBT self-help programs in two studies, group psychodynamic therapy in one study, short-term intervention for alcohol problems in one study, attention modification program in one study, and individual cognitive therapy in one study. The medications that were used in these studies included the MAOI phenelzine in one study, MAOI moclobemide in one study, SSRI fluoxetine in one study, SSRI paroxetine in two studies, SSRI escitalopram in one study, benzodiazepine clonazepam in one study, and either an SSRI or SNRI in one study. All combination therapy studies employed different combinations of psychological and pharmacological interventions. Four studies showed a benefit of combined psychological intervention and pharmacotherapy, but combination therapy or comparators were different across studies, and there were five studies that did not show a benefit of combination therapy. The certainty of combination therapy is very low, and the balance between desirable and undesirable effects cannot be determined. Below is an overview of the individual studies:

***NICE Guideline***

Blanco et al. (2010)^3^ conducted a randomized controlled trial with 128 patients with social anxiety disorder by comparing group CBT and phenelzine (combination treatment group: 32 patients) with group CBT (34 patients), phenelzine (35 patients), and placebo (27 patients). After 12 weeks of treatment, social anxiety symptoms on the LSAS improved significantly in the combination therapy and phenelzine groups compared with the placebo group but not compared with the group CBT group. Combination treatment was significantly superior to monotherapy alone. The posttreatment response and remission rates were highest in the combination therapy group (response rate: 71.9%; remission rate: 46.9%), followed by the phenelzine group (response rate: 54.3%; remission rate: 22.9%), group CBT group (response rate: 47.1%; remission rate: 8.8%), and placebo group (response rate: 33.3%; remission rate remission rate: 7.4%). The dropout rates were 37.1% in the phenelzine group, 35.3% in the group CBT group, 28.1% in the combination therapy group, and 18.5% in the placebo group. In a network meta-analysis in the NICE guideline, the mean post-intervention social anxiety symptom rating scale score was 1.69 standard deviations lower in the combination therapy group compared with the treatment waitlist group (Standardized mean difference from the network meta-analysis [SMD^N^] = -1.69).

Craske et al. (2011)^4^ conducted an RCT of 1004 patients with anxiety-related disorders (generalized anxiety disorder, panic disorder, social anxiety disorder, and PTSD) by comparing a group who received treatment based on patient preference (74 with social anxiety disorder, with patient choice of medication [SSRIs or SNRIs as first choice], supported computerized CBT, or a combination of both) and a group who received standard treatment (58 with social anxiety disorder). After 6 months of treatment, social anxiety symptoms on the SPIN improved significantly more in the group that received treatment based on patient preference than in the group that received standard treatment. Details of post-treatment response and remission rates were unavailable, but response rates were significantly higher in the group that received treatment based on patient preference compared with the standard treatment group, with no group differences in remission rates. The dropout rate was 9.5% in the patient preference-based treatment group and 15.5% in the standard treatment group. In the network meta-analysis in the NICE guideline, the mean post-intervention social anxiety symptom rating scale score was 0.48 standard deviations lower in the group that received patient preference-based treatment compared with the waitlist group (SMD^N^ = -0.48).

Davidson et al. (2004)^5^ conducted an RCT in 295 patients with social anxiety disorder by comparing group CBT plus fluoxetine (combination therapy group: 59 patients), group CBT plus placebo (59 patients), group CBT (60 patients), and placebo (58 patients). After 14 weeks of treatment, social anxiety symptoms on the Brief Social Phobia Scale significantly improved in all treatment groups compared with the placebo group, with no differences between treatment groups and no advantage of combination therapy. Post-treatment response rates were 54.2% in the combination group, 51.7% in the group CBT group, 50.9% in the fluoxetine group, 50.8% in the placebo combination group, and 31.7% in the placebo group. The treatment dropout rates were 40.0% in the placebo group, 31.6% in the fluoxetine group, 28.8% in the combination group, and 20.0% in the group CBT group. In the network meta-analysis in the NICE guideline, the mean social anxiety symptom rating scale score after the intervention was 0.95 standard deviations lower in the combination therapy group compared with the treatment waitlist group (SMD^N^ = -0.95).

Prasko (2003)^6^ conducted an RCT in 81 patients with social anxiety disorder by comparing group CBT plus moclobemide (combination therapy group; 22 patients), group CBT plus placebo (placebo group; 24 patients), and moclobemide (20 patients). After 3 months of treatment, social anxiety symptoms on the LSAS significantly improved in all treatment groups, and the combination group was the most effective. Details of post-treatment response, remission, and dropout rates were unavailable. In the network meta-analysis in the NICE guideline, the mean post-intervention social anxiety symptom rating scale score was 1.23 standard deviations lower in the combined therapy group compared with the treatment waitlist group (SMD^N^ = -1.23).

Knijnik et al. (2008)^7^ conducted a randomized controlled trial of group psychodynamic therapy plus clonazepam (29 patients) *vs*. clonazepam (29 patients) in 58 patients with social anxiety disorder. After 12 weeks of treatment, there was no significant difference in social anxiety symptoms on the LSAS between the two groups, indicating no benefit of the combination treatment for social anxiety symptoms. Post-treatment response rates were 79.3% in the combination therapy group and 53.6% in the clonazepam group. Remission rates were 10.3% and 3.6% (based on the LSAS) in the combination and phenelzine groups, respectively, and 31% and 25% (based on CGI-I), respectively. Dropout rates were 3.4% in the combination group and 17.2% in the clonazepam group. In the NICE guideline network meta-analysis, the mean post-intervention social anxiety symptom rating scale score was 1.28 standard deviations lower in the combination therapy group compared with the treatment waitlist group (SMD^N^ = -1.28).

***Surveillance report (2017)***

Book et al. (2013)^8^ conducted an RCT by comparing a short-term intervention for alcohol problems plus paroxetine (44 participants) *vs*. paroxetine (39 participants) in 83 patients with social anxiety disorder and drinking problems. After 22 weeks of treatment, social anxiety symptoms on the LSAS significantly improved in both groups, with no difference between groups and no advantage of the combination treatment. Post-treatment response and remission rates were unavailable. Dropout rates were 6.8% in the combination therapy group and 23.1% in the paroxetine group. Drinking behavior as a coping strategy significantly improved in both groups, with no differences between groups.

Gingnell et al. (2016)^9^ conducted an RCT in 48 patients with social anxiety disorder by comparing a supported computer-based CBT self-help program with escitalopram (combination therapy group: 24 patients) *vs*. a placebo group (24 patients). After 12 weeks of treatment, social anxiety symptoms on the LSAS significantly improved in the combination therapy group compared with the placebo group. Post-treatment response rates were 66.7% in the combination group and 33.3% in the placebo group. The dropout rate was 0% in the combination therapy group and 4.2% in the placebo group.

Khedmatgozar et al. (2012)^10^ conducted an RCT in 33 patients with social anxiety disorder by comparing an attention modification program plus paroxetine group (11 patients), an attention modification program group (11 patients), and a paroxetine group (11 patients). After 8 weeks of treatment, social anxiety symptoms on the SPIN significantly improved in the combination therapy group compared with the attention modification program group but not compared with the other monotherapy groups, indicating no advantage of combination therapy. Post-treatment response and remission rates were unavailable. Treatment dropout rates were 18.2% each in the combination therapy and paroxetine groups and 9.1% in the attention modification program group.

Nordahl et al. (2016)^11^ conducted an RCT by comparing 102 patients with social anxiety disorder in a combined individual cognitive therapy and paroxetine group (26 patients), individual cognitive therapy group (24 patients), paroxetine group (26 patients), and placebo group (26 patients). After 12 weeks of treatment, when compared with the placebo group, social anxiety symptoms on the LSAS significantly improved in both the combination therapy group and the individual cognitive therapy group, but not significantly in the paroxetine group. The combination therapy and individual cognitive therapy groups also showed significant improvement compared with the paroxetine group, with no difference between the combination therapy and individual cognitive therapy groups, indicating no advantage of the paroxetine combination therapy over individual CBT alone. Post-treatment remission rates were 45.0% in the combination therapy group, 68.2% in the individual cognitive therapy group, 23.8% in the paroxetine group, and 4.3% in the placebo group. Dropout rates were 23.1% in the combination therapy group, 8.3% in the individual cognitive therapy group, 19.2% in the paroxetine group, and 11.5% in the placebo group.

References for Supplementary Information CQ3:

1. National Institute for Health and Care Excellence (NICE). Social anxiety disorder: recognition, assessment and treatment (Clinical guideline [CG159]): Leicester (UK): British Psychological Society; 2013 Available from: http://guidance.nice.org.uk/CG159.

2. National Institute for Health and Care Excellence (NICE). Surveillance report 2017 – Social anxiety disorder: recognition, assessment and treatment (2013) NICE guideline CG159: Leicester (UK): British Psychological Society; 2017 Available from: https://www.nice.org.uk/guidance/cg159/resources/surveillance-report-2017-social-anxiety-disorder-recognition-assessment-and-treatment-2013-nice-guideline-cg159-4484818333/chapter/Surveillance-decision?tab=evidence.

3. Blanco C, Heimberg RG, Schneier FR, Fresco DM, Chen H, Turk CL, et al. A placebo-controlled trial of phenelzine, cognitive behavioral group therapy, and their combination for social anxiety disorder. Arch Gen Psychiatry. 2010;67(3):286-95.

4. Craske MG, Stein MB, Sullivan G, Sherbourne C, Bystritsky A, Rose RD, et al. Disorder-specific impact of coordinated anxiety learning and management treatment for anxiety disorders in primary care. Arch Gen Psychiatry. 2011;68(4):378-88.

5. Davidson JR, Foa EB, Huppert JD, Keefe FJ, Franklin ME, Compton JS, et al. Fluoxetine, comprehensive cognitive behavioral therapy, and placebo in generalized social phobia. Arch Gen Psychiatry. 2004;61(10):1005-13.

6. Prasko J, Kosova J, Paskova B, Klaschka J, Seifertova D, Sipek J. Pharmacotherapy and/or cognitive-behavioral therapy in the treatment of social phobia: Control study with two year follow up. Ces Slov Psychiatr. 2003;99:106-8. [in Czech]

7. Knijnik DZ, Blanco C, Salum GA, Moraes CU, Mombach C, Almeida E, et al. A pilot study of clonazepam versus psychodynamic group therapy plus clonazepam in the treatment of generalized social anxiety disorder. Eur Psychiatry. 2008;23(8):567-74.

8. Book SW, Thomas SE, Smith JP, Randall PK, Kushner MG, Bernstein GA, et al. Treating individuals with social anxiety disorder and at-risk drinking: phasing in a brief alcohol intervention following paroxetine. J Anxiety Disord. 2013;27(2):252-8.

9. Gingnell M, Frick A, Engman J, Alaie I, Björkstrand J, Faria V, et al. Combining escitalopram and cognitive-behavioural therapy for social anxiety disorder: randomised controlled fMRI trial. Br J Psychiatry. 2016;209(3):229-35.

10. Khedmatgozar H, Birashk B, Ashayeri H, Farid AA. Comparing the effectiveness of paroxetine, Attention Modification Program and combination of both on improving Social Anxiety Symptoms. Basic Clinl Neurosci. 2012;3:36-44.

11. Nordahl HM, Vogel PA, Morken G, Stiles TC, Sandvik P, Wells A. Paroxetine, Cognitive Therapy or Their Combination in the Treatment of Social Anxiety Disorder with and without Avoidant Personality Disorder: A Randomized Clinical Trial. Psychother Psychosom. 2016;85(6):346-56.

**2. Second-line treatment**

There is a lack of evidence and no recommendation about whether a combination of pharmacotherapy and psychotherapy is recommended when initial treatment is ineffective or causes a partial response. Further research is needed.

The Guideline Development Committee offered the following suggestions as expert opinions without evidence.

- If the patient does not show adequate improvement after receiving an appropriate series of individual CBT, then we suggest the addition (combination) of pharmacotherapy with individual CBT. However, benefits and harms of pharmacotherapy should be fully explained to the patient and implemented in accordance with the patient’s preferences.
- If patients do not show adequate improvement after 10-12 weeks of treatment with an SSRI, then we suggest the addition (combination) of individual CBT with an SSRI. However, the addition of individual CBT can only be proposed after providing a sufficient explanation of the cost of attending a medical institution where individual CBT can be administered and the cost burden.

**3. Treatment for children under 18 years old**

The treatment of patients younger than 18 years of age was not examined in the guideline. Young patients tend to experience more adverse events with SSRIs than adults. Therefore, they should be treated more cautiously with pharmacotherapy.

**4. Changes in treatment options due to comorbidities**

There is a lack of evidence and no recommendation about whether a combination of pharmacotherapy and psychotherapy is recommended for comorbidities.
